# An unknown source of reactor radionuclides in the Baltic Sea revealed by multi-isotope fingerprints

**DOI:** 10.1038/s41467-021-21059-w

**Published:** 2021-02-05

**Authors:** Jixin Qiao, Haitao Zhang, Peter Steier, Karin Hain, Xiaolin Hou, Vesa-Pekka Vartti, Gideon M. Henderson, Mats Eriksson, Ala Aldahan, Göran Possnert, Robin Golser

**Affiliations:** 1 Department of Environmental Engineering, Technical University of Denmark, DTU Risø Campus, Roskilde, Denmark; 2 Northwest Institute of Nuclear Technology, Xi’an, China; 3 Faculty of Physics, Isotope Physics, University of Vienna, Vienna, Austria; 4 Environmental Radiation Surveillance, Radiation and Nuclear Safety Authority, Helsinki, Finland; 5 Department of Earth Sciences, University of Oxford, Oxford, UK; 6 Department of Health, Medicine and Caring Sciences, Linköping University, Linköping, Sweden; 7 Department of Radiation Protection, Swedish Radiation Safety Authority, Stockholm, Sweden; 8 Department of Geology, United Arab Emirates University, Al Ain, United Arab Emirates; 9 Tandem Laboratory, Uppsala University, Uppsala, Sweden

**Keywords:** Environmental sciences, Ocean sciences, Marine chemistry

## Abstract

We present an application of multi-isotopic fingerprints (i.e., ^236^U/^238^U, ^233^U/^236^U, ^236^U/^129^I and ^129^I/^127^I) for the discovery of previously unrecognized sources of anthropogenic radioactivity. Our data indicate a source of reactor ^236^U in the Baltic Sea in addition to inputs from the two European reprocessing plants and global fallout. This additional reactor ^236^U may come from unreported discharges from Swedish nuclear research facilities as supported by high ^236^U levels in sediment nearby Studsvik, or from accidental leakages of spent nuclear fuel disposed on the Baltic seafloor, either reported or unreported. Such leakages would indicate problems with the radiological safety of seafloor disposal, and may be accompanied by releases of other radionuclides. The results demonstrate the high sensitivity of multi-isotopic tracer systems, especially the ^233^U/^236^U signature, to distinguish environmental emissions of unrevealed radioactive releases for nuclear safeguards, emergency preparedness and environmental tracer studies.

## Abstract (web summary)

Anthropogenic activities lead to the accumulation of radioactive substances in the environment. Here the authors use multi-isotopic fingerprints of uranium and iodine to discover a previously unknown source of reactor uranium in the Baltic Sea, likely sourced from a Swedish nuclear facility.

## Introduction

^236^U (*t*_½_ = 2.34 × 10^7^ years) is an isotope of uranium that is produced by thermal neutron capture of ^235^U via (n, γ)-reactions and through ^238^U (n, 3n) ^236^U reactions with fast neutrons. Even though a small amount of ^236^U (~35 kg) occurs naturally in the Earth’s crust, ^236^U is (by mass) the largest secondary product created in nuclear reactors, estimated to be ~10^6^ kg^1^. ^236^U is therefore a sensitive tracer of deliberate or accidental leakage from the nuclear fuel/waste cycle^2–5^. The known sources of reactor ^236^U, i.e., deliberate releases from the two European reprocessing plants at La Hague, France (LH), and Sellafield, UK (SF) since 1950s, can be traced throughout the North Atlantic and the Arctic water currents^6^. Emissions from other known sources of reactor ^236^U, e.g., the Springfield nuclear facility and the Fukushima accident, are negligible^5,7^.

A significant amount of ^236^U (estimated at >1000 kg) was also delivered to the Earth’s surface environments from the global fallout of atmospheric nuclear weapons testing in the 1950s and 1960s^8^. This ubiquitous fallout signature can make identification of sources of reactor ^236^U challenging because of methodological difficulties in distinguishing the source of ^236^U^9^. In addition, the ^236^U/^238^U ratio does not provide source information because of the prevalence of ^238^U in nature.

Reactor ^236^U can be differentiated from fallout ^236^U because these sources have different and characteristic ^233^U/^236^U ratios due to different nuclear production mechanisms. ^233^U was mostly produced during nuclear weapons testing by fast neutrons via ^235^U (n, 3n) ^233^U reactions or directly by ^233^U-fueled devices, whereas almost no ^233^U is produced in thermal nuclear power reactors or reprocessing plants^10^. Recently ^233^U measurements at environmental levels have become possible with advanced accelerator mass spectrometry^10^.

The representative ^233^U/^236^U atomic ratio of global fallout from atmospheric nuclear weapons testing was suggested to be (1.40 ± 0.15) × 10^−2^ ^9^. This is several orders of magnitude higher than the ^233^U/^236^U atomic ratio in nuclear reactors, e.g., 1 × 10^−7^–1 × 10^−6^ in LH discharges^11^, which agrees well with reactor model calculations^12^. In the Irish Sea, an average ^233^U/^236^U atomic ratio of (0.12 ± 0.01) × 10^−2^ has been measured^9^, reflecting a dominant reactor signal released from SF. The use of the ^233^U/^236^U atomic ratio helps to deconvolve the origin of ^236^U based on the characteristic ^233^U/^236^U fingerprint from different source terms. In addition, the combination of ^236^U with other radionuclides, e.g., ^129^I, can be useful to trace the transport of ^236^U from specific source points, e.g., releases from LH and SF^13–16^.

The Baltic Sea is a highly polluted sea, with anthropogenic radionuclides demanding specific attention because of the risk to ecosystem and humans from radioactivity in the environment. It receives radionuclides from global fallout, discharges from the two European reprocessing plants, releases from the Chernobyl accident, and from any other local sources. In this study, we use a novel combination of three anthropogenic radionuclides—^233^U, ^236^U, and ^129^I—to identify a previously unknown local source of radionuclide pollution to the Baltic Sea.

## Results and discussion

### Study area and sampling

**Fig. 1:**
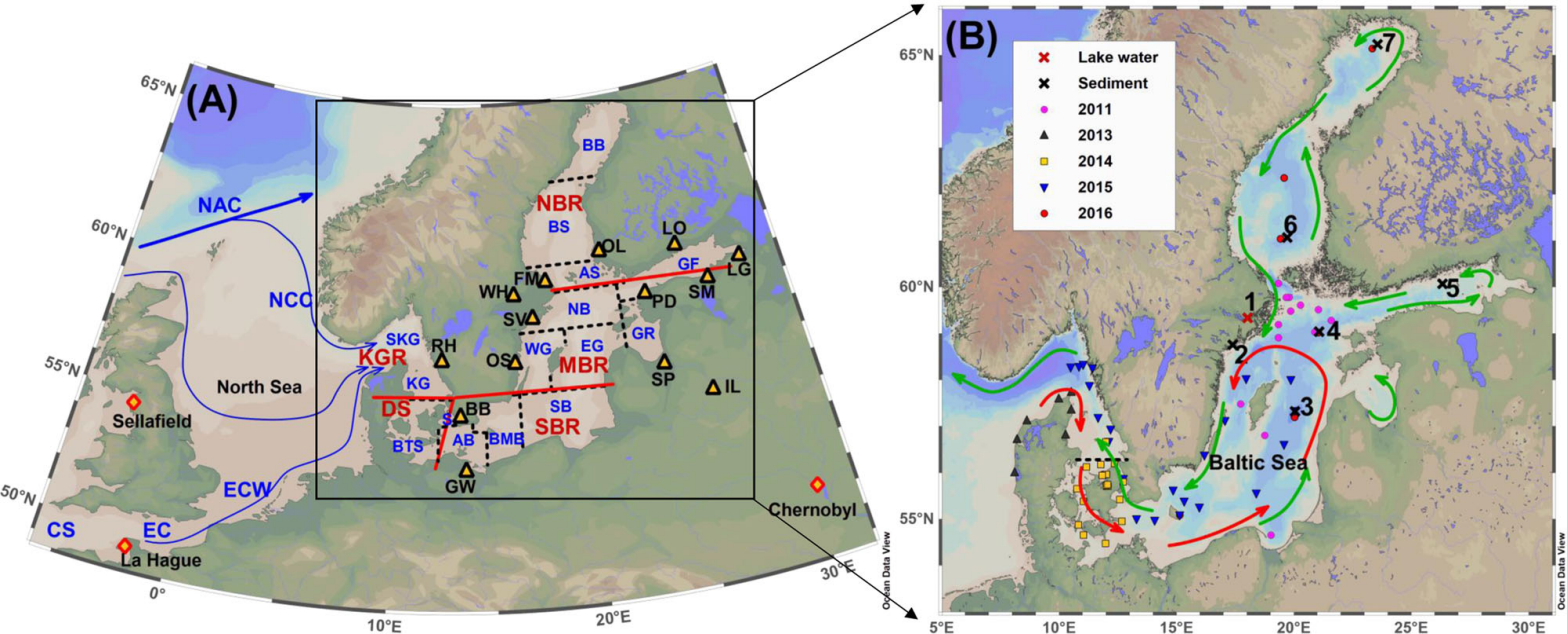
Study region and sampling map. Overview of schematic circulation water mass in North Sea-Baltic Sea region (**A**) and sampling stations in this work as well as nuclear installations around the Baltic Sea (**B**). The symbols in **A** are CS Celtic Sea, EC English Channel, ECW English Channel Waters, NAC North Atlantic Current, NCC Norwegian Coastal Current, BB Bothnian Bay, BS Bothnian Sea, AS Archipelago and Åland Sea, GF Gulf of Finland, NB Northern Baltic Proper, WG Western Gotland Basin, EG Eastern Gotland Basin, GR Gulf of Riga, SB Sourth Baltic Proper, BMB Bornholm Basin, AB Arkona Basin, S The Sound, BTS Belt Sea, KG Kattegat, SKG Skagerrak, KGR Kattegat–Skagerrak region including the Kattegat, Skagerrak and Danish west coast nearby the North Sea, DS Danish Straits including the Belt Seas and the Sound, SBR South Baltic Sea region including Arkona Basin, Borholm Basin, and South Baltic Proper, MBR Middle Baltic Sea region including Northern Baltic Proper, Western Gotland Basin, Eastern Gotland Basin and Gulf of Riga, and NBR North Baltic Sea region including Archipelago and Åland Sea, Bothnian Sea and Bothnian Bay) Nuclear installations including: RH Ringhals NPP, BB Barseback NPP, GW Greifswald NPP, OS Oskarshamn NPP, SV Studsvik AB site, WH Westinghouse Electric Sweden AB, FM Forsmark NPP, OL Olkiluoto NPP, LO Loviisa NPP, LG Leningrad NPP, IL Ignalina NPP, SM Sillamäe site, PD Paldiski site, SP Salaspils research reactor. The stations marked with cross in **B** are either lake water or sediment samples (1—Lake Mälaren water; 2—Studsvik sediment; 3—sediment BY15; 4—sediment LL17; 5—sediment LL3a; 6—sediment EB1; 7—sediment CVI), all the other samples are seawaters collected in different years during 2011–2016 as marked with different symbols. Red arrows refer to bottom water movement and green arrows refer to surface water movement.

The Baltic Sea is a landlocked intracontinental sea in Northern Europe with about 80 million inhabitants in the surrounding states and constitutes one of the largest brackish water environments on Earth^17^. The water exchange of this large brackish estuarine-like water mass with the Kattegat and the North Sea takes place through the narrow and shallow Danish Straits (Fig. 1). The driving force for the water circulation is freshwater surplus from river runoff, estimated at 473 km^3^ per year, together with “recycled” North Sea inflowing water as Baltic outflow that sum to a total water exchange rate of 753 km^3^ per year^18^. A mean residence time for the 21,721 km^3^ Baltic water volume^19^ was estimated to be 29 years, which is equivalent to a “half-life” for the water volume of 20 years^18^.

In the investigation presented here, water and sediment samples were collected from the Baltic Sea and related water masses including the western Danish coast, from 2011 to 2016 (Supplementary Tables 1 and 2). The majority of water samples are from the surface (0–5 m depth), with a few samples from deep water, and one lake water from the Lake Mälaren, which receives downstream discharges from a nuclear fuel fabrication facility (Westinghouse) in Sweden and finally drains into the Baltic Sea. In addition to the Baltic Sea water, we analyzed sediment samples to assess the accumulation trend of the isotopes in the Baltic Sea. A more detailed description of the study area and samples can be found in the “Methods” section.

To facilitate the presentation of results and related discussion, we grouped the sampling locations into five geographical regions (Fig. 1) in the Baltic Sea including (1) KGR: Kattegat–Skagerrak region including the Kattegat, Skagerrak and Danish west coast nearby the North Sea; (2) DS: Danish Straits including the Belt Seas and the Sound; (3) SBR: South Baltic Sea region including Arkona Basin, Borholm Basin, and South Baltic Proper; (4) MBR: Middle Baltic Sea region including Northern Baltic Proper, Western Gotland Basin, Eastern Gotland Basin, and Gulf of Riga; and (5) NBR: North Baltic Sea region including Archipelago and Åland Sea, Bothnian Sea and Bothnian Bay.

### Spatial pattern of ^236^U concentration and ^236^U/^238^U and ^233^U/^236^U atomic ratios

The measured ^236^U/^238^U atomic ratios (Supplementary Tables 1 and 2) vary within (5–52) × 10^−9^, with the higher ratios in the central and northern parts of the Baltic Sea and lower ratios in the western parts (Danish Straits, Kattegat, Skagerrak, and Danish west coast). The highest value reported here is sixfold greater than the average value found in the North Sea in 2010 ((7.6 ± 3.7) × 10^−9^)^20^.

**Fig. 2:**
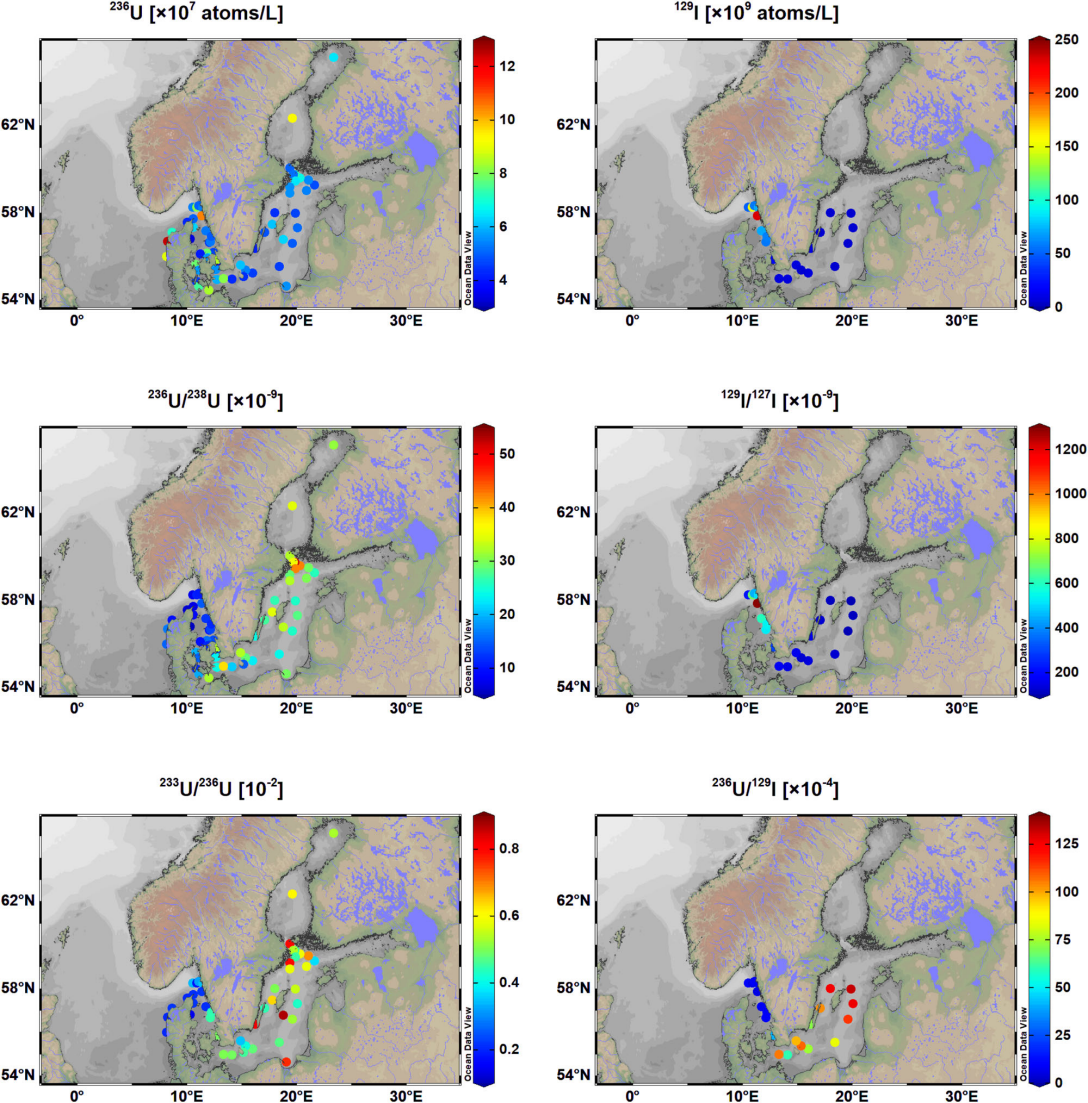
Results of anthropogenic radionuclides. Distribution of ^236^U and ^129^I concentrations, and ^236^U/^238^U, ^129^I/^127^I, ^233^U/^236^U, and ^236^U/^129^I atomic ratios in the Baltic Sea surface water during 2011–2016.

The spatial patterns (Fig. 2) suggest a general decline of ^236^U with distance from higher values in the North Sea which is expected to be dominated by discharges from LH and SF. However, high ^236^U concentrations ((6–9) × 10^7^ atom/l) are observed in the surface water of the Bothnian Sea and Borthnian Bay, which are comparable to values ((3–10) × 10^7^ atom/l) in the central North Sea^20^. Compared to the Kattegat–Skagerrak region, the average ^236^U/^238^U atomic ratio in the middle and north Baltic region increases by a factor of 3, from (10 ± 3) × 10^−9^ to (32 ± 7) × 10^−9^. This pattern of increasing in ^236^U/^238^U ratio highlights an additional, likely local, source of ^236^U in the Baltic Sea^7^.

^233^U/^236^U atomic ratios obtained here are in the range of (0.14–0.87) × 10^−2^, with the lowest ^233^U/^236^U atomic ratios in the western parts of the Baltic, including the Danish coast, and the highest ratios in the central Baltic Sea. As the typical ^233^U/^236^U ratio for global fallout is (1.4 ± 0.1) × 10^−2^^9^, the high ^233^U/^236^U in the central Baltic Sea could indicate either strong influence of global fallout or addition from a local source.

### Distribution of ^129^I concentration, ^129^I/^127^I and ^236^U/^129^I atomic ratios

The measured ^129^I concentrations ((3–232) × 10^9^ atom/l) and ^129^I/^127^I atomic ratios ((101–1286) × 10^−9^) in the seawater collected in this work show comparable values and distribution trends as observed in an earlier investigation^21^, with the highest values in the North Sea-Skagerrak–Kattegat, decreasing values toward the Sound and relatively constant values in the Baltic Proper. The distributions of ^129^I concentrations and ^129^I/^127^I atomic ratios indicate that the major source of ^129^I in the Baltic Sea are marine discharges from the two nuclear reprocessing plants at LH and SF. The water mass pathways from these plants have been shown to contain appreciable amounts of ^129^I along the passage to the Baltic Sea^21^.

Aldahan et al. ^22^ reported that the average concentration of ^129^I in the rivers around the Baltic Sea was 3.9 × 10^8^ atom/l, which suggested some minor contribution of ^129^I from riverine water to the Baltic Sea. The ^129^I concentrations obtained in this work show a larger gradient (two orders of magnitude) compared to the ^236^U concentrations (15-fold) along the Baltic Sea. ^236^U/^129^I ratios are within the range of (5–133) × 10^−4^ and indicate a reversed geographical distribution compared to ^129^I concentration and ^129^I/^127^I atomic ratio (Fig. 2).

### Potential sources of uranium and iodine in the Baltic Sea

Five different sources of uranium and iodine in the Baltic Sea are:

1. Natural ocean water, with salinity of 35‰, which contains ~60 µg/l ^127^I, 3 µg/l ^238^U, but negligible ^129^I, ^236^U, and ^233^U.
2. Natural freshwater with salinity <1‰, negligible ^129^I, ^236^U, and ^233^U, and significantly lower ^127^I and ^238^U than seawater (0.05–10 µg/l for both nuclides).
3. Global fallout from atmospheric nuclear weapons testing, with negligible ^127^I and ^238^U, an average ^233^U/^236^U atomic ratio of (1.40 ± 0.15) × 10^−2^, and a surface geographical distribution pattern for ^236^U and ^233^U similar to that of other actinides (e.g., Pu) from global fallout^23^. Earlier studies have estimated ^236^U concentration (up to 1.4 × 10^8^ atom/l peaking in 1960s) in surface water of the North Sea to be related to global fallout, which may have been partly masked by discharges from the nuclear reprocessing of LH and SF^24,25^. In the Baltic Sea, with an average depth of 55 m, the dilution by vertical dispersion is limited, and a ten times higher concentration is expected for the same inventory, which might mimic higher input. The ^233^U/^236^U atomic ratio of the global fallout contribution is expected to be constant after 1980 when all countries stopped aboveground nuclear bomb tests. Concentration of ^236^U in river runoff is expected to have reduced over the decades, while the ^233^U/^236^U atomic ratio stays constant.
4. Marine discharges from European nuclear fuel reprocessing plants (including mainly SF and LH), with known ^236^U and ^129^I source functions^24,26^, but negligible amounts of ^127^I and ^238^U. This source dominates the ^236^U and ^129^I budget of marine water entering the Skagerrak from the North Sea. Compared to ^236^U, almost no ^233^U is produced in thermal nuclear reactors, and thus ^233^U should also be absent from marine discharges of the reprocessing plants.
5. The Chernobyl accident. Pu from Chernobyl has been found in fallout over central Europe^27^ and, as Pu and U are refractory elements transported similarly by atmospheric dispersion, Chernobyl ^236^U should have been deposited following a similar pattern as Pu isotopes. Consequently, a Chernobyl signal of ^236^U may be present in river runoff and marine waters. Based on the present understanding of the production mechanisms of ^233^U, it is expected that Chernobyl fallout is not a significant contributor of ^233^U in this context.

Waters entering the Baltic Sea from the North Sea have ^236^U/^238^U and ^233^U/^236^U atomic ratios set by the balance of reprocessing discharge and global fallout^9,20^. As they are distributed in the Baltic and mix with waters from various rivers, ratios can be altered by addition from local sources of ^236^U and ^233^U (and minor ^238^U in river waters). Removal of uranium from Baltic water will not alter the ratios. The increase in ^236^U/^238^U observed within the Baltic Sea points clearly to a local source of this anthropogenic radionuclide.

### ^236^U source identification via binary mixing

**Fig. 3:**
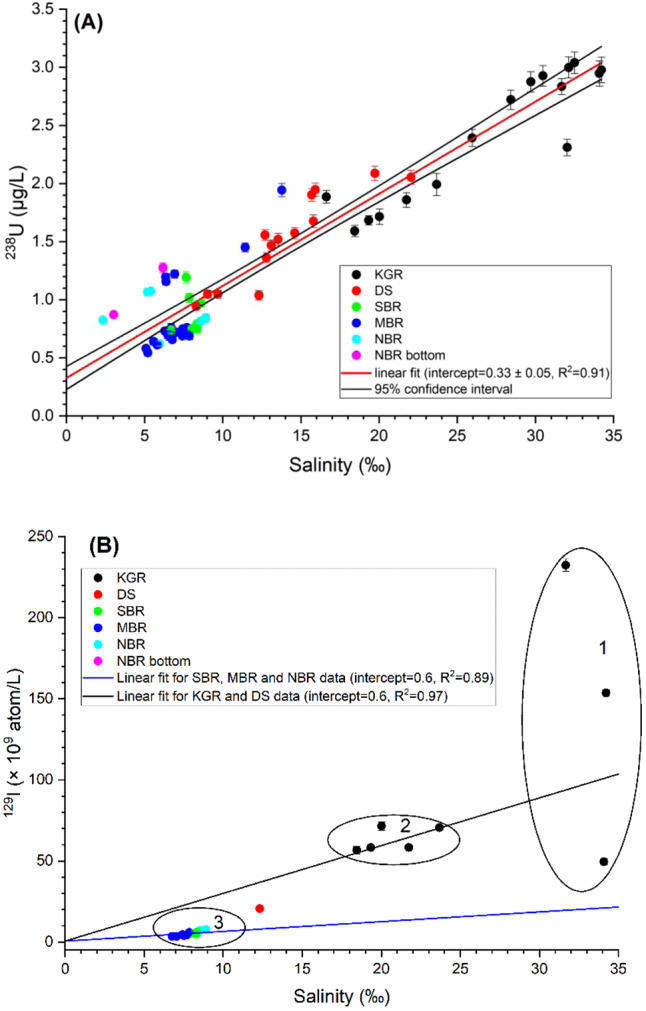
Variations of ^238^U and ^129^I with salinity. ^238^U (**A**) and ^129^I (**B**) concentrations vs. salinity in the Baltic Sea. KGR Kattegat–Skagerrak region including the Kattegat, Skagerrak and Danish west coast nearby the North Sea, DS Danish Straits including the Belt Seas and the Sound, SBR South Baltic Sea region including Arkona Basin, Borholm Basin, and South Baltic Proper, MBR Middle Baltic Sea region including Northern Baltic Proper, Western Gotland Basin, Eastern Gotland Basin and Gulf of Riga, NBR North Baltic Sea region including Archipelago and Åland Sea, Bothnian Sea and Bothnian Bay. The zones (1–3) in **B** refer to dominant water mass: 1—North Sea-North Atlantic water, 2—Kattegat–Skagerrak water, and 3—Baltic Sea water. The intercept for linear regression line of ^129^I concentration vs. salinity was constrained to 0.6 × 10^9^ atom/l according to the reported minimum ^129^I concentration in the Baltic river water^22^. Uncertainties are expanded uncertainties using a coverage factor of *k* = 1.

The concentration of ^238^U (Fig. 3A) demonstrates a strong positive correlation (*R*^2^ = 0.91) with salinity. The intercept corresponds to the average riverine input with a ^238^U concentration of 0.33 ± 0.05 µg/l, which falls in the range (0.2–0.7 µg/l) of ^238^U for some rivers in the Baltic Sea region^28^. We will use the typical value 0.4 µg/l in the following calculations. There is more scatter in the ^238^U concentration for low salinities, which might be attributed to differences in regional riverine input. ^129^I also shows a general positive linear correlation with salinity demonstrated by two mixing lines for the western (KGR-DS, *R*^2^ = 0.89) and interior (SBR-MBR-NBR, *R*^2^ = 0.97) region (Fig. 3B). The scatter at the high salinity end can be attributed to the mixing of ^129^I enriched North Sea coast water with ^129^I depleted North Atlantic water in the Kattegat–Skagerrak region. The ^238^U and ^129^I trends with salinity suggest that their concentrations in the Baltic Sea are mainly controlled by the saline water input from the North Sea via Kattegat–Skagerrak, mixing with fresh waters in the basin.

Both the ^236^U/^238^U and ^236^U/^129^I atomic ratios increase with the decreasing salinity as waters mix in the interior of the Baltic Sea. The ^236^U/^238^U ratio increases by a factor of 3, while the ^236^U/^129^I ratio increases greater than an order of magnitude from an average of (8 ± 2) × 10^−4^ in the Kattegat–Skagerrak region, corresponding to reprocessing derived ^236^U and ^129^I, to 1 × 10^−2^ in the central Baltic Sea. Both ratios indicate addition of ^236^U from a local source. If the source does not contain any ^129^I, the tenfold increase in ^236^U/^129^I suggests that ca. 90% of ^236^U in the central Baltic Sea is from local sources. If the source does contain ^129^I, the portion of ^236^U derived locally must be still larger.

**Fig. 4:**
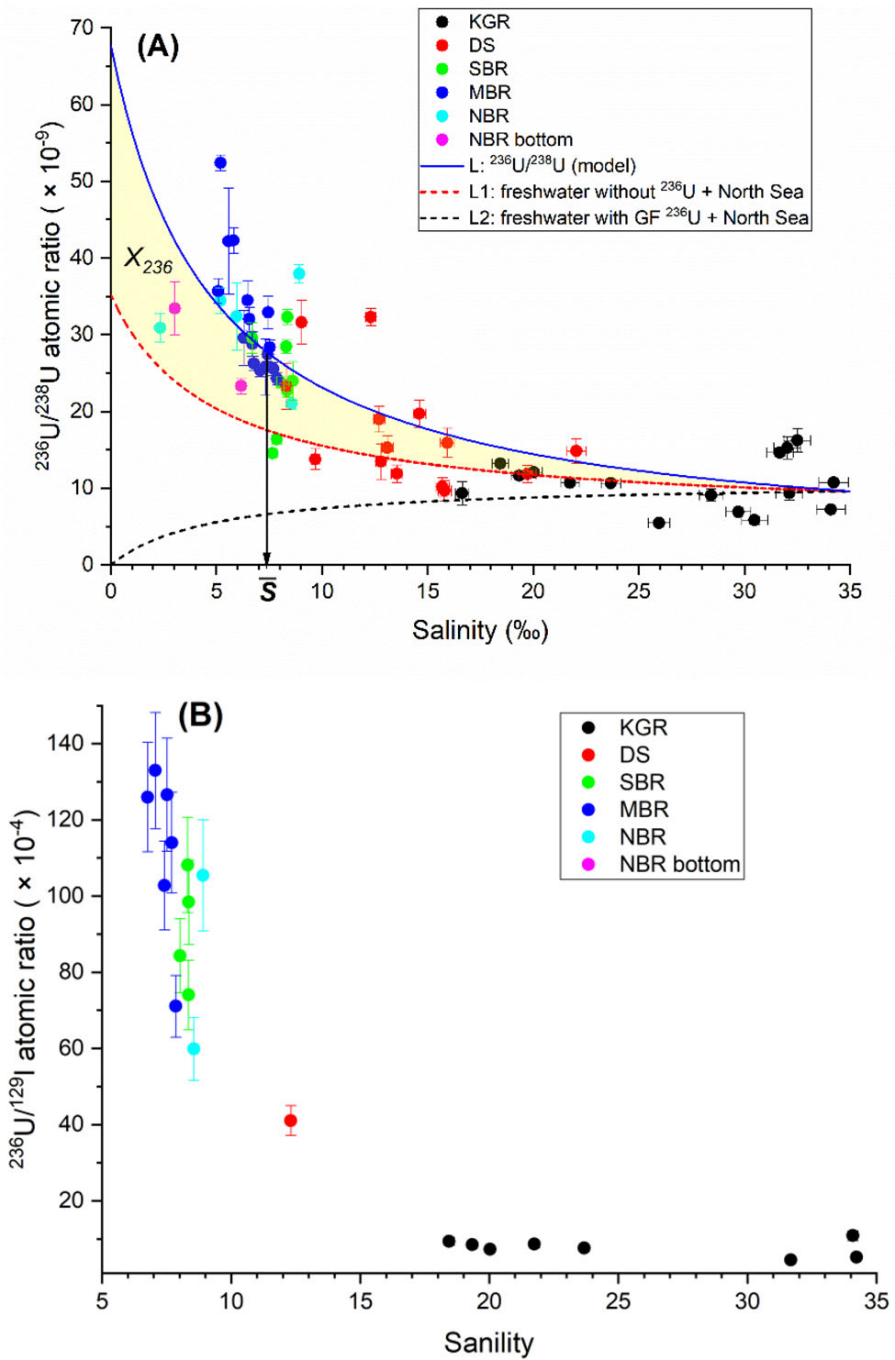
Variations of ^236^U/^238^U and ^236^U/^129^I with salinity. ^236^U/^238^U atomic ratio (**A**) and ^236^U/^129^I atomic ratios (**B**) vs. salinity. KGR Kattegat–Skagerrak region including the Kattegat, Skagerrak and Danish west coast nearby the North Sea, DS Danish Straits including the Belt Seas and the Sound, SBR South Baltic Sea region including Arkona Basin, Borholm Basin, and South Baltic Proper, MBR Middle Baltic Sea region including Northern Baltic Proper, Western Gotland Basin, Eastern Gotland Basin, and Gulf of Riga, NBR North Baltic Sea region including Archipelago and Åland Sea, Bothnian Sea, and Bothnian Bay, L (blue solid line) the best-fit binary mixing line between the North Sea water and a freshwater end member with salinity = 0, ^238^U = 0.4 µg/l, ^236^U/^238^U atomic ratio = (6.79 ± 0.75) × 10^−8^, and ^236^U = (6.87 ± 0.76) × 10^7^ atom/l, L1 (black dashed line) the binary mixing line between the North Sea water and an assumed freshwater end member containing no ^236^U (salinity = 0, ^238^U = 0.4 µg/l, ^236^U/^238^U atomic ratio = 0 and ^236^U = 0, L2 (red dashed line) the binary mixing line between the North Sea water and the best-fit freshwater end member with salinity = 0, ^238^U = 0.4 µg /L, ^236^U = (3.56 ± 0.39) × 10^7^ atom/l, and ^236^U/^238^U atomic ratio = (3.52 ± 0.39) × 10^−8^. The area marked in yellow represents the estimated excess mass of ^236^U in the Baltic Sea (*X*_236_), average salinity $\bar S$ = 7.36‰. Uncertainties are expanded uncertainties using a coverage factor of *k* = 1.

To understand the source terms of ^236^U in the Baltic Sea, a binary mixing model is applied with two respective end members representing ^236^U input from the North Sea and freshwater input via river runoff. Parameters for the first end member representing the North Sea water entering from the west Baltic Sea are well defined by previous studies (Supplementary Table 3)^20,29^. The deviation of the observed ^236^U/^238^U atomic ratio in the binary mixing (line L1, Fig. 4A) of the North Sea water and an assumed freshwater end member containing no ^236^U (neither ^233^U) from the best-fit model L reflects additional ^236^U sources besides North Sea water. The spatial distribution of deviations in the ^236^U/^238^U atomic ratio enable determination of the location of the additional ^236^U source (Supplementary Fig. 2). The distribution pattern is compatible with the introduction of additional riverine ^236^U input from the north Baltic region, which has most river runoff.

Nevertheless, it is challenging to define the ^236^U/^238^U ratio of the riverine input to the Baltic because a component of global fallout may still be present in runoff from the land surface. The ^236^U/^238^U and ^236^U/^129^I ratios cannot be used to determine the extent to which the excess ^236^U is from global fallout or an additional, previously undiscovered, source that has directly released ^236^U to the Baltic Sea.

### Application of ^233^U/^236^U atomic ratio for ^236^U source identification

**Fig. 5:**
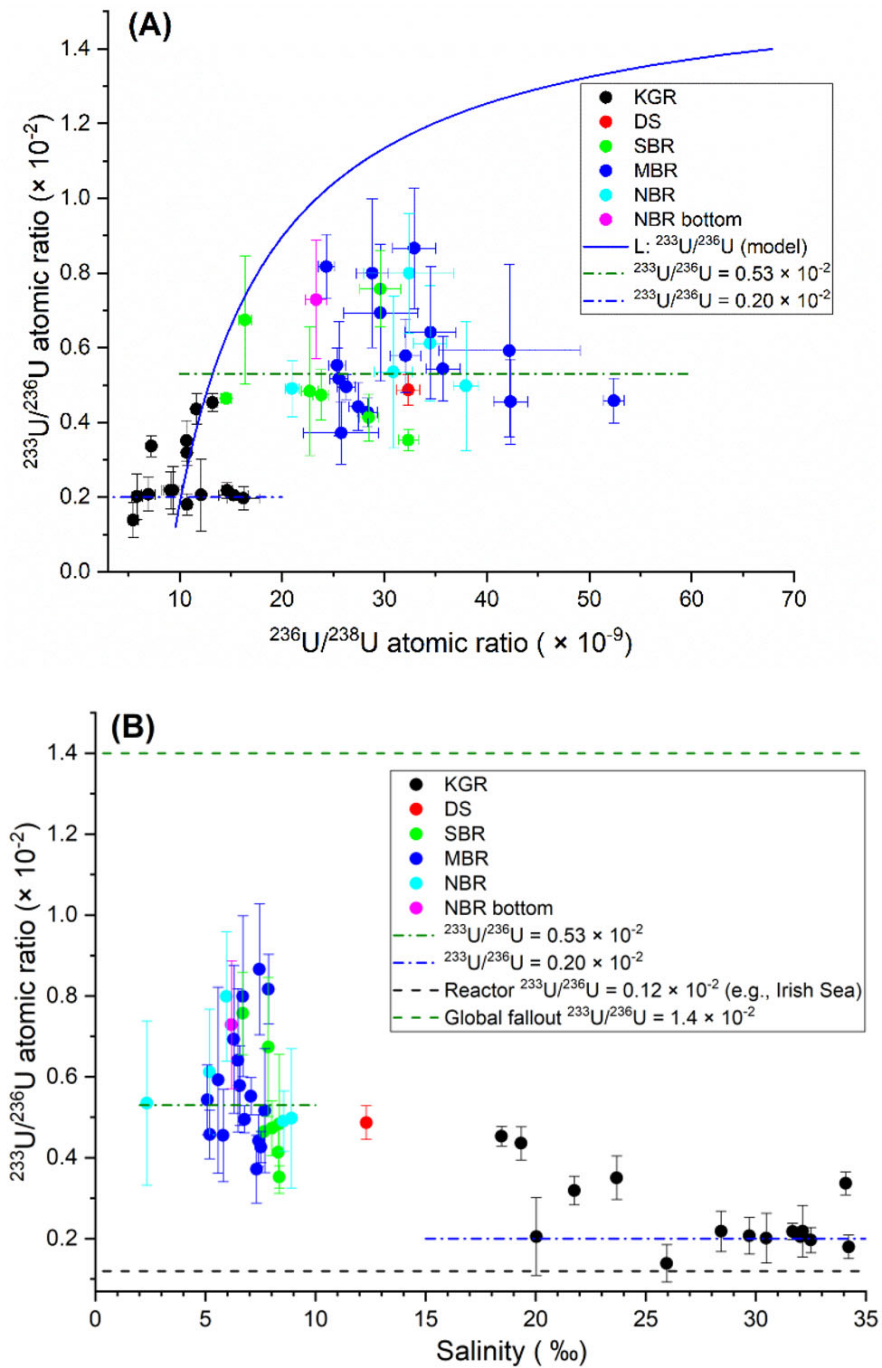
Results of ^233^U/^236^U. ^233^U/^236^U atomic ratio vs. ^236^U/^238^U atomic ratio (**A**) and salinity (**B**). KGR Kattegat–Skagerrak region including the Kattegat, Skagerrak and Danish west coast nearby the North Sea, DS Danish Straits including the Belt Seas and the Sound, SBR South Baltic Sea region including Arkona Basin, Borholm Basin, and South Baltic Proper, MBR Middle Baltic Sea region including Northern Baltic Proper, Western Gotland Basin, Eastern Gotland Basin and Gulf of Riga, NBR North Baltic Sea region including Archipelago and Åland Sea, Bothnian Sea, and Bothnian Bay, L (blue solid line) the best-fit binary mixing line between the North Sea water and a freshwater end member with salinity = 0, ^238^U = 0.4 µg/l, ^236^U/^238^U atomic ratio = (6.79 ± 0.75) × 10^−8^, and ^236^U = (6.87 ± 0.76) × 10^7^ atom/l. Uncertainties are expanded uncertainties using a coverage factor of *k* = 1.

If we assume that the excess ^236^U originates only from global fallout, the ^236^U/^238^U atomic ratio of the riverine input in the best-fit binary mixing is (6.79 ± 0.75) × 10^−8^ (line L, Fig. 4A). However, there is a clear deviation of the observation from the model for ^233^U/^236^U atomic ratios (Fig. 5A). A subgroup of samples from the Kattegat–Skagerrak reveal a relatively stable ^233^U/^236^U atomic ratio of 0.20 × 10^−2^ (blue dash-dotted line in Fig. 5) independent of ^236^U/^238^U and salinity. This behavior can be explained by assuming an end member of North Sea water with ^233^U/^236^U atomic ratio = 0.20 × 10^−2^ (a mixed signal of global fallout plus nuclear reprocessing) and salinity 35‰, which is mixed with natural uranium or water with neither ^236^U nor ^233^U. This feature shows the notable impact of nuclear reprocessing from SF and LH in the region.

On the other hand, a cluster of samples with the majority from the south, middle and north Baltic Sea region, representative for a large part of the Baltic surface water and with median salinity (6.92 ± 0.29)‰, show a typical ^233^U/^236^U atomic ratio of (0.53 ± 0.03) × 10^−2^ (the green dash-dotted line in Fig. 5). This cluster lies significantly below the binary mixing model L, indicating an additional local ^236^U sources besides the global fallout, which is characterized by low ^233^U/^236^U atomic ratio. A low ^233^U/^236^U atomic ratio is typical for releases from nuclear reactors, thereby we assume such a reactor-related source of ^236^U with negligible ^233^U in the following.

About two-thirds of the anthropogenic uranium observed in the middle and north Baltic Sea region seems to originate from this additional local source (Eq. (1)), indicating a strong contribution of ^236^U without ^233^U, i.e., from a thermal nuclear reactor ^236^U.

1 $${{R}}_{\mathrm{s}} = \frac{{{\mathrm{N}}_{233,{\mathrm{f}}} + {\mathrm{N}}_{233,{\mathrm{r}}}}}{{{\mathrm{N}}_{236,{\mathrm{f}}} + {\mathrm{N}}_{236,{\mathrm{r}}}}} = \frac{{{\mathrm{N}}_{236,{\mathrm{f}}} \cdot {{R}}_{\mathrm{f}} + {\mathrm{N}}_{236,{\mathrm{r}}} \cdot {{R}}_{\mathrm{r}}}}{{{\mathrm{N}}_{236,{\mathrm{f}}} + {\mathrm{N}}_{236,{\mathrm{r}}}}} = \frac{{{{R}}_{\mathrm{f}} + {\mathrm{N}}_{236,{\mathrm{r}}}/{\mathrm{N}}_{236,{\mathrm{f}}} \cdot {{R}}_{\mathrm{r}}}}{{1 + {\mathrm{N}}_{236,{\mathrm{r}}}/{\mathrm{N}}_{236,{\mathrm{f}}}}}$$

where *R*_s_, *R*_f_, and *R*_r_ represent, respectively, the ^233^U/^236^U atomic ratio of the Baltic seawater, global fallout, and nuclear reactor; N_233, f_ and N_233,r_ refer to the atomic number of ^233^U from global fallout and nuclear reactor, respectively; N_236, f_ and N_236, r_ refer to the atomic number of ^236^U from global fallout and nuclear reactor, respectively. Therefore, $\frac{{{\mathrm{N}}_{236,{\mathrm{r}}}}}{{{\mathrm{N}}_{236,{\mathrm{f}}}}} = \frac{{{{R}}_{\mathrm{f}} - {{R}}_{\mathrm{s}}}}{{{{R}}_{\mathrm{s}} - {{R}}_{\mathrm{r}}}}$. With *R*_s_ = (0.53 ± 0.03) × 10^−2^, *R*_f_ = 1.4 × 10^−2^, and *R*_r_ = 0.12 × 10^−2^ (the Irish Sea ratio), we calculate the ^236^U contribution from our assumed reactor source to be 2.1 ± 0.2 times that of global fallout.

**Fig. 6:**
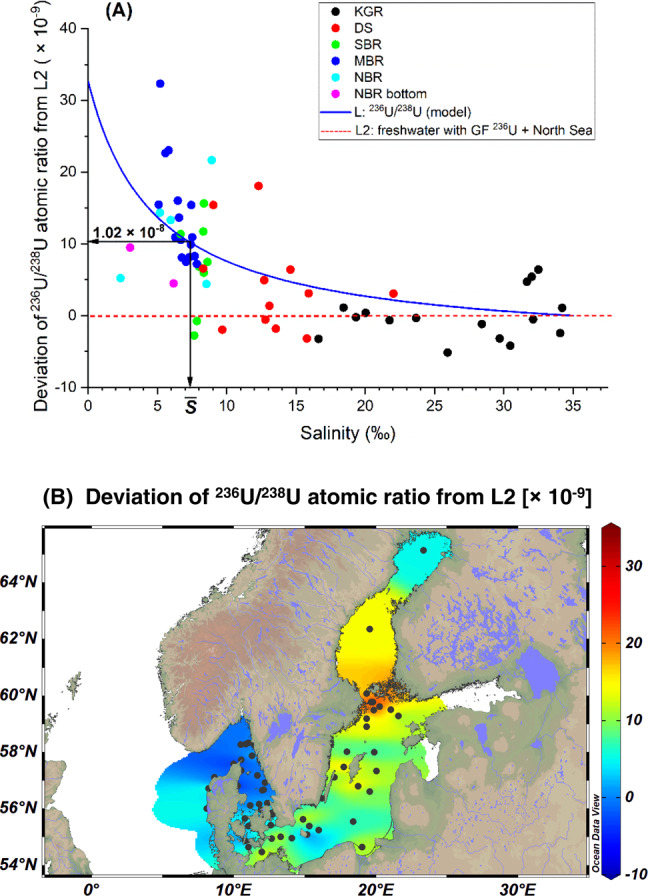
Deviation of ^236^U/^238^U from L2. Deviations of ^236^U/^238^U atomic ratio from binary mixing line L2 (**A**) and their respective geographical distribution on the map (**B**). KGR Kattegat–Skagerrak region including the Kattegat, Skagerrak and Danish west coast nearby the North Sea, DS Danish Straits including the Belt Seas and the Sound, SBR South Baltic Sea region including ArkonaBasin, Borholm Basin, and South Baltic Proper, MBR Middle Baltic Searegion including Northern Baltic Proper, Western Gotland Basin, EasternGotland Basin and Gulf of Riga, NBR North Baltic Sea region includingArchipelago and Åland Sea, Bothnian Sea, and Bothnian Bay. Average salinity $\bar S$ = 7.36‰. Uncertainties are expanded uncertainties using a coverage factor of *k* = 1.

To locate this additional reactor ^236^U source, we apply another binary mixing line L2 (Fig. 4A) of the North Sea water with riverine water, the latter carrying global fallout that accounts for 1/(1 + 2.1) of the average ^236^U concentration of our samples in the Baltic Sea. Thus, the freshwater end member is characterized by salinity = 0, ^238^U = 0.4 µg/l, ^236^U = (3.56 ± 0.39) × 10^7^ atom/l, which is calculated to match the ^233^U/^238^U atomic ratio ((1.70 ± 0.18) × 10^−10^) for the cluster of samples from SBR, MBR, and NBR at the media salinity of (6.92 ± 0.29)‰ (Supplementary Fig. 2). The resultant ^236^U/^238^U atomic ratio of the freshwater end member is (3.52 ± 0.39) × 10^−8^. The excesses of the ^236^U/^238^U atomic ratio from the mixing curve L2 and their spatial distribution are shown in Fig. 6. The data indicate that the extra reactor ^236^U source input is not from places where salinity is particularly low or where there are rivers, but in the middle and north basins of the Baltic Sea which is probably linked to direct releases of ^236^U into these locations.

### Properties of the unknown ^236^U source

To narrow down the possible sources of the excess ^236^U, ^236^U inventories and fluxes need to be estimated. It should be noted this calculation is a first-order approximation based only on our data on surface waters from a multi-year survey. A precise interpretation will require more data, and to account for many different effects such as vertical distribution of ^236^U in the Baltic water columns, inter-annual variation in distribution pattern and on the scavenging of uranium into the sediment (especially in the anoxic regions).

The existence of an additional source of anthropogenic ^236^U in the Baltic Sea is indicated by the difference between the models L2 and L (Fig. 6). The amount of ^236^U required to explain this difference can be calculated by the following approximation and with the uncertainty estimated according to Müller^30^:

2 $$X_{236} = \, {\int}_0^{35} \left( {{\mathrm{L}}(S) - {\mathrm{L}}2(S)} \right)\left[ {{\,}^{238}{\mathrm{U}}(S)} \right]\frac{{dV\left( S \right)}}{{dS}}dS\\ \approx \, \left( {{\mathrm{L}} - {\mathrm{L2}}} \right)\left( {\bar S} \right)\left[ {{\,}^{238}{\mathrm{U}}\left( {\bar S} \right)} \right]V_{BS} = 200 \pm 47{\mathrm{g}}$$

where *X*_236_ is the excess mass of ^236^U in the Baltic Sea, *S* is the salinity, $\left[ {{\,}^{238}{\mathrm{U}}\left( S \right)} \right]$ is the ^238^U concentration corresponding to *S* taken from Fig. 3A. $\bar S$ is the average salinity of the Baltic Sea. $\overline S$ was taken as 7.36‰ based on the reported mean salinity of the Baltic Sea during 1902–1998^31^. Our data from SBR, MBR, and NBR, which comprise the bulk of Baltic Sea water, show an average of salinity of 7.06‰ and a median of 6.92%, comparable to the reported value. $\left[ {{\,}^{238}{\mathrm{U}}\left( {\bar S} \right)} \right]$ is 0.9 µg/l. *V*_*BS*_ is the volume of the Baltic Sea (21,721 km^3^)^19^ and $({\mathrm{L}} - {\mathrm{L2}})(\bar S)$ = (1.02 ± 0.24) × 10^−8^ is the difference of the model curves at the average salinity. The approximation in the formula is possible because in the models L and L2, the ^236^U concentration is a linear function of *S*. Therefore, 200 ± 47 g of ^236^U is from the additional reactor source.

This calculation is a snapshot in time based on the uranium isotope ratios and salinity. While uranium concentrations in water may be altered in the partly anoxic Baltic Sea by precipitation of inorganic U(IV) or binding to organics in the sediment, the uranium isotopic ratios will only change by mixing of difference sources. Total salinity is slightly affected by precipitation (rain and snow) and evaporation (net balance 63 km^3^ per year)^32^, which may, be neglected at the present level of precision. Large intrusions of the North Sea water can change salinity patterns and introduce anthropogenic uranium from the North Sea. These intrusions add up to 5.2 × 10^9^ metric tons of salt^33^, which is about 3% of the salt inventory of the Baltic^32^. The spatial pattern may not be constant throughout a multi-year survey, nevertheless, a minor change in the calculation is expected as we use only the average salinity for our estimate.

Taking into account that the ratio between the additional source and global fallout is N_236,r_/N_236,f_ = 2.1, it suggests that 95 ± 22 g of ^236^U is related to global fallout introduced into the Baltic Sea directly or via riverine input. It is estimated that a total inventory of 1000 kg of anthropogenic ^236^U was distributed via global fallout mainly on the Northern Hemisphere^7^. Considering the surface area of the Baltic Sea of 3.77 × 10^5^ km^2^ (without the catchment area) in comparison to the Northern Hemisphere (i.e., 2.55 × 10^8^ km^2^), the total ^236^U deposition from direct global fallout is estimated as 1.5 kg. However, if considering the 29-year mean residence time (equivalent to 20-year half-life) of Baltic seawater, then most of the deposited 1.5 kg ^236^U was transported out of the region after 60 years (i.e., three half-lives), leaving behind ~0.19 kg. In addition, some ^236^U fraction from global fallout might be removed from the water body and incorporated into the Baltic sediment^34^. Therefore, the above estimation of 95 ± 22 g remaining ^236^U in the Baltic seawater from global fallout seems plausible, considering the uneven distribution of global fallout.

If we include the Baltic catchment area (1.64 × 10^6^ km²) in the calculation, the input of global fallout ^236^U in the Baltic region can be up to 8 kg (1.5 kg in seawater + 6.4 kg in catchment area). However, only a small fraction of the particle associated ^236^U deposited on land can be leached and transported to the Baltic Sea through river runoff. If we assume this fraction accounts for 10% of the 6.4 kg of ^236^U deposited in the catchment, the total amount of global fallout ^236^U in the Baltic Sea might be about 0.64 + 1.5 = 2.14 kg.

Emissions from the Chernobyl accident may contribute additional ^236^U to the Baltic Sea, but it is difficult to quantify. Nuclear dumping and/or nuclear installations around the Baltic countries are also possible source candidates. As marked in Fig. 1, there are many nuclear installations in surrounding Baltic countries, but there is very limited documentation with poor, unreleased or missing data about the ^233^U and ^236^U release records from these installations (Supplementary Table 4)^11^. Data for ^236^U are available from Westinghouse during 1998–2017, with a total reported release of 1.06 × 10^6^ Bq of ^236^U, equal to 0.44 g. In addition, we measured one lake water sample collected in Lake Mälaren (Supplementary Table 2), which receives waste discharges from the Westinghouse facility and finally drains into the Baltic Sea. The results show that the ^236^U/^238^U ratios is at the level of 2 × 10^−8^, which is comparable with the seawater samples collected in the central Baltic Sea. The lake water shows a ^233^U/^236^U atomic ratio of (0.18 ± 0.05) × 10^−2^, a signature of reactor material.

The amount of ^236^U released from the Westinghouse installation (0.44 g) is negligible compared to the above estimated 280 g of the unknown reactor source in the Baltic Sea. For the Lake Mälaren, the ^238^U concentration was measured to be 1.5 ± 0.1 µg/l in this work, together with a flux of 166 m^3^/s, it means an input of 0.1 g per year of ^236^U, which is negligible also.

Another candidate for the additional source may be reactor fuel, dumped into the Baltic. The atomic ratio of ^236^U/^238^U can be as high as 1 × 10^−2^ in conventional nuclear reactors, which would require only 27 kg of dumped/dissolved fuel (a commercial nuclear reactor contains ~100,000 kg of fuel). ^235^U enrichment in reactor fuel is 3% for light-water reactors, up to 10% for thermal gas-cool reactors and up to 20% for fast reactors^35^. The concentration will be even higher in the core of a nuclear reactor for marine applications, where enriched or highly enriched ^235^U is used; Russian submarine reactors were reported to contain 50–200 kg of ^235^U^36^. The former Soviet Union (USSR) was accused of dumping radioactive waste in the Baltic Sea, but it is not possible to assess the dumped amount^37,38^.

The geographical distribution of ^236^U/^238^U atomic ratio in surface seawater of central Baltic Sea shows high values nearby the Swedish coast close to Stockholm, which is within ~100 km of a nuclear research company Studsvik AB, Nyköping that has been in operation since 1950s. It was reported that during 1959 and 1961, 64 tons of radioactive waste with total radioactivity of 14.8 GBq were dumped into the coastal area nearby Studsvik^39^. The aquatic discharges of radionuclides (except ^3^H) from Stusvik into the Baltic Sea in 1999–2010 were reported to be 0.45 TBq with the majority consisting of ^90^Sr, ^137^Cs, ^60^Co, and ^134^Cs^40^. Our measurements on some sediment samples from the Studsvik area show very high ^236^U content ((2.02 ± 0.12) × 10^13^ atom/kg), which is three orders of magnitude higher than sediment collected from the North Baltic Sea region (Supplementary Table 2). The ^233^U/^236^U atomic ratio ((0.36 ± 0.05) × 10^−2^) for the Studsvik sediment clearly indicates a higher contribution of reactor input compared to the other five sediments collected in the Baltic Sea with ^233^U/^236^U ratios between 0.59 × 10^−2^ to 0.83 × 10^−2^.

Even though the release of ^236^U from Studsvik is not well documented due to its low specific radioactivity, it is not surprising that waste discharges from Studsvik contain ^236^U. The high ^236^U levels in the sediment samples measured most likely originate from scavenging of waterborne ^236^U from liquid waste discharges by particles into the sediment. Waste dumping/discharges in the Studsvik area are our most plausible candidate for the excess ^236^U in the Baltic Sea.

### Outlooks for future study

The radiological risk associated with ^233^U, ^236^U, and ^129^I observed in this work is negligible due to their low specific activities and radiotoxicities. However, the observed unknown ^236^U reactor source may be an indication of leakage from a previously unrecognized (or unreported) additional radioactive source in the Baltic Sea, e.g., disposed nuclear waste in the seabed. Such source could potentially contain ^137^Cs and many other radionuclides imposing high radiological risks. Recent studies of the distribution of ^137^Cs inventories in the Baltic Sea indicated that ^137^Cs deposited in surface sediments is not permanently buried, but may be re-suspended and re-deposited by currents, bioturbation, or anthropogenic activities^41^. This leads us to suggest that radioactive release from such a source although currently low, might become more significant in the future with climate and environmental changes (e.g., sea level, temperature, and pH) in the Baltic Sea. It will be important to further understand the sources of anthropogenic radioisotopes in the Baltic regions, so that prediction and monitoring can prevent any associated radiological risk in the future. Further observation and forensic work will be needed to tighten the constraints in the binary mixing models, provide clear source terms and radiation risk assessment.

## Methods

### Detailed description of the study area and sampling

The Baltic Sea features three major basins, the Bothnian Bay, the Bothnian Sea, and the Baltic Proper. The two northerly basins (Bothnian Bay and Bothnian Sea) are characterized by low salinity water mass (1–3‰ and 3–7‰, respectively) and weak vertical salinity stratification, although strong thermoclines usually develop during the summer^42^. The Bothnian Sea represents a large reservoir of brackish water mass that can be divided into two layers blocked by a weak halocline around a depth of 60 m. The long-term circulation of the Bothnian Sea water is dominated by an estuary circulation, where the bottom dense waters can be traced as surface water in the Baltic Proper^43^. The Baltic Proper is the largest basin in the Baltic Sea, permanently stratified in the central part with a strong halocline around a depth of 75 m separating the surface water (salinity 7–8‰) from the deep water (salinity 9–20‰) and a long-term cyclonic current circulation pattern^44^. Water exchange in the Baltic Proper happens through renewing of the deep water during extreme inflow events from the Kattegat. The water mass circulation is further associated with outflow of surface water to the Kattegat and inflow of fresher surface waters from the Bothnian Sea, the Gulf of Finland and the Gulf of Riga (Fig. 1).

Water samples analyzed in the present investigation were collected on different cruises during 2011–2016. Samples of 2011 were obtained from the Baltic GEOTRACES Process Study on board research vessel R/V Oceania. Samples from 2013 to 2014 were collected through the environmental monitoring program for Helsinki Commission (HELCOM). Samples from 2015 were collected on board the research vessel Argos, operated by the marine division of the Swedish Metrological and Hydrological Institute. Samples from 2016 were obtained from the Radiation and Nuclear Safety Authority (STUK), Finland, through sampling cruise COMBINE 2 on the research vessel R/V Aranda. One lake water sample from Lake Mälaren (in Sweden: 59.33 °N, 18.04 °E) was also sampled for the radioisotope analyses, as this lake receives downstream discharges from a nuclear fuel fabrication facility (Westinghouse) in Sweden, which finally drains into the Baltic Sea.

Five surface (0–2 cm) sediments in the middle and north parts of Baltic region were collected (Fig. 1 and Supplementary Table 2) during the COMBINE 2 cruise in 2016. One sediment sample collected outside Studsvik AB in Bergasundet, Bergas strait (58.75 °N, 17.40 °E) in 2014, which was obtained by pooling 25 sediment plugs (0–10 cm) and homogenized at Swedish Radiation Safety Authority (SSM). The Bergasundet, Bergas strait was the drainage area of the nuclear research facility (Studsvik AB). Details of the sampling campaigns and location of samples are summarized in Supplementary Tables 1 and 2 and Fig. 1.

### Standards and reagents

Uranium standard solution (1.000 g/l in 2 M HNO_3_) purchased from NIST (Gaithersburg, MD) was used after dilution as a standard for the ICP-MS measurement to quantify ^238^U in seawater. All reagents used in the experiment were of analytical reagent grade and prepared using ultra-pure water (18 MΩ cm). UTEVA resin (100–150 µm particle size) was purchased from Triskem International, Bruz, France and packed in 2-ml Econo-Columns (0.7 cm i.d. × 5 cm length, Bio-Rad Laboratories Inc., Hercules, CA) for the chemical purification of uranium isotopes. The in-house ^236^U standards Vienna-KkU (^236^U/^238^U = (6.89 ± 0.32) × 10^−11^)^1^ and Vienna-US8 (^236^U/^238^U = (1.01 ± 0.05) × 10^−8^)^45^ diluted by ion (U/Fe = 1:30) were used to monitor the accuracy of the AMS measurement. Five standard samples (3 × Vienna-US8 and 2 × Vienna-KkU) were measured with a batch of around 30 environmental samples. The Vienna-KkU also serve as machine blank for the detection of ^233^U by AMS.

### Analytical methods for determination of ^238^U, ^236^U, ^233^U, ^127^I, and ^129^I

The concentration of ^238^U and ^127^I in seawater was measured by ICP-MS (X Series^II^, Thermo Fisher Scientific, Waltham, MA) after 10–50 times dilution with 0.5 M HNO_3_ and 0.1 M NH_3_·H_2_O, respectively. The ICP-MS instrument was equipped with an Xt-skimmer core and a concentric nebulizer under hot plasma conditions. The typical operational conditions of the instrument have been given elsewhere^46^. Indium (as InCl_3_) as an internal standard and 0.5 M HNO_3_ as a washing solution between consecutive assays were applied for ^238^U, and caesium (as CsCl) as an internal standard and 0.1 M NH_3_·H_2_O as a washing solution were used for ^127^I.

The radiochemical method for ^233^U and ^236^U separation from seawater was applied according to Qiao et al.^47^. Each seawater sample (0.8–10 l) were filtrated with filter paper (Munktell 00 K, particle retention 5–6 µm) to remove large particles and then acidified to pH 2 with concentrated HNO_3_. Purified FeCl_3_ solution (0.05 g/ml of Fe) was added to a final Fe concentration of 0.1 g/l. The sample was vigorously stirred with air bubbling for 5–10 min in order to decompose carbonate complexes. In total, 10% NH_3_·H_2_O was slowly added to adjust the pH to 8–9 for the co-precipitation of U with Fe(OH)_3_. The precipitate was allowed to settle for 0.5–1 h in order to decant most of the supernatant. The sample slurry was centrifuged at 3000 × *g* for 5 min and the supernatant was discarded. The final residue was dissolved with 15 ml of 3 M HNO_3_ and the solution was loaded onto a 2-ml UTEVA column which was preconditioned with 20 ml of 3 M HNO_3_. The UTEVA column was rinsed with 40 ml of 3 M HNO_3_, followed by 20 ml of 6 M HCl. Uranium absorbed on the column was eluted with 10 ml of 0.025 M HCl. The flow rate for the chromatographic separation was controlled manually to 1.0–1.5 ml/min.

A 100-µl aliquot of U eluate from the column separation was taken for measurement of ^238^U by ICP-MS to evaluate the chemical yields by comparison with ICP-MS analysis on diluted seawater samples. The ^238^U content measured in the eluate was also used for blank subtraction to calibrate the actual ^236^U/^238^U and ^233^U/^238^U atomic ratios^47^. The remaining fraction was used to prepare target for the AMS measurement of ^236^U/^238^U and ^233^U/^236^U. For sediments, 5–10 g of each dried sample was ashed overnight at 450 °C in a muffle oven and leached with 100 ml of aqua regia on a hotplate for 30 min at 150 °C and then 2 h at 200 °C. A 100-µl aliquot leachate was taken for direct measurement of ^238^U by ICP-MS, which was used to calculate the ^238^U concentration in the sediment sample. The remaining leachate was processed following the same procedure (i.e., Fe(OH)_3_ co-precipitation and UTEVA column separation) as for seawater samples.

The AMS measurement was carried out at the 3-MV tandem accelerator facility Vienna Environmental Research Accelerator (VERA) at the University of Vienna, Austria^9,10,48^. To summarize, U, which is extracted as UO^−^ from a cesium sputter ion source, has to pass a first mass separation stage before it is injected into a tandem accelerator. For the analysis of actinides, the accelerator is operated at a terminal voltage of 1.65 MV and a rather high helium pressure in the terminal stripper is used to suppress molecular background^49^. The relatively high stripper gas pressure causes losses of a significant fraction of U^3+^ ions to angular scattering and change exchange outside of the stripper assembly. This gives an effective stripping yield of around 21% for the charge state 3+^50^, which is selected by the subsequent 90° analysing magnet. The combination of the analysing magnet with a Wien filter, an electrostatic analyzer, and a second 90° magnet, efficiently suppresses isotopic background on the high-energy side. Possible isotopic background is mainly caused by ^235^U and ^232^Th that are injected into the accelerator as ^235^U^16^O^1^H and ^232^Th^16^O^1^H, respectively. At the end of the AMS set-up, a Bragg-type ionization chamber is installed in order to detect and identify the remaining ions.

^238^UH^3+^ which escapes destruction in the stripping process gives a background to mass 239, 3+ lower than ^238^UH^3+^/^238^U^3+^ = 10^−14^. A similar suppression is expected for ^235^UH^3+^/^235^U^3+^, which suggests an instrumental background for ^236^U below ^235^UH^3+^/^238^U^3+^ = 10^−16^, which is negligible compared to the background of real ^236^U extracted from the ion source. The mass 239, 3+ background is monitored for every sputter sample. The situation is different for ^233^U^3+^, where the potentially interfering molecular isobar is ^232^ThH^3+^. In fact, an even higher intensity of these molecules was found from a similar ion source^51^. As thorium is a different chemical element, the behavior of hydride during stripping cannot be predicted from uranium ions. However, because thorium is only a trace element in our sputter samples, much less suppression than for ^235^UH^3+^ would be sufficient to render ^232^ThH^3+^ insignificant as background for ^233^U^3+^. For quality control, ^232^Th^3+^ is monitored for all sputter samples, which is extracted as ^232^ThO^−^. Though substantial rate above 100 kHz (too high for quantification by our detector) were observed in some cases, no correlation with the mass 233, 3+ count rates were found. This suggests that ^232^ThH^3+^ is also sufficiently supressed by the high stripper gas pressure.

A detection efficiency of 2 × 10^−4^ for environmental samples and a detection limit for ^236^U/U below 10^−14^ has been reported for the VERA set-up^10^. Because of the small relative mass difference (ca. 1%), fractionation effects between^233^U and ^236^U are negligible, therefore a detection efficiency comparable to ^236^U is assumed for ^233^U. In samples with low ^236^U content, e.g., procedure blanks, the uncertainty of ^236^U/^238^U atomic ratio measured by AMS is mainly attributed to the counting statistics, while for environmental samples the precision usually is limited by the reproducibility of multiple measurements which is taken into account in the overall uncertainty of 1–5% as well. Due to the low count rates of environmental ^233^U, the uncertainty of the ^233^U/^238^U atomic ratio is dominated by counting statistics of ^233^U. As the ^238^U content in the sample determined by ICP-MS was used for blank correction of the atomic ratios measured by AMS, the overall uncertainty of the blank corrected values presented in Supplementary Table 1 is therefore a combination of the corresponding AMS and ICP-MS uncertainties calculated by error propagation.

For the determination of ^129^I in seawater, 100 ml of sample was transferred into separation funnels. In total, 2.0 mg of ^127^I carrier (prepared using iodine crystal purchased from Woodward company, USA, with a ^129^I/^127^I ratio of 2 × 10^−14^), 500 Bq of ^125^I^−^ tracer, and 0.5 ml of 0.5 M Na_2_S_2_O_5_ solution were added to the funnel, and then the pH of the solution was adjusted to 1–2 using 3 M HNO_3_ to convert all iodine species to iodide. With addition of 20–50 ml chloroform (CHCl_3_) and 2–5 ml 1.0 M NaNO_2_, iodide was oxidized to I_2_ and extracted to CHCl_3_ phase by shaking. The extraction procedure was repeated three times to extract all iodine. The CHCl_3_ phases were combined to a new funnel, 20 ml H_2_O and 0.3–0.5 ml 0.05 M Na_2_SO_3_ solution was added to the funnel to reduce I_2_ in chloroform phase to iodide and back-extracted iodine into water phase. This extraction and back extraction processes were repeated once for further purification.

The separated iodine (in iodide form) in a small volume (5–7 ml) was transferred to a centrifuge tube, 1.0 ml of 0.5 M AgNO_3_ solution and 1 ml of 3.0 M HNO_3_ were added to form AgI precipitate. The AgI precipitate was separated using centrifugation at 2300 × *g* for 3–5 min, and washed in sequence using 10 ml 3 M HNO_3_ and two times of 10 ml deionized water to remove possibly formed Ag_2_SO_3_ and Ag_2_SO_4_ which are soluble in acidic solution. The precipitate was transferred to a 1.5 ml centrifuge tube. ^125^I in the precipitate was measured using a NaI gamma detector for calculating the chemical yield of iodine. The prepared AgI precipitate in small tube was dried at 70 °C and weighed. The dried precipitate was ground to fine powder and mixed with five times by mass of niobium powder (325 mesh, Alfa Aesar, Ward Hill, MA), which was finally pressed into a copper holder using a pneumatic press. ^129^I/^127^I atomic ratios in the prepared targets were measured by the 5 MV AMS system at the Tandem Laboratory, Uppsala University. The standard used in the measurement was prepared by dilution of ^129^I standard (NIST-SRM-4949c) and mixed with ^127^I carrier to a ratio of ^129^I/^127^I of 1.0 × 10^−11^. All samples, blanks, and standards were measured for six cycles and 5 min per sample in each cycle. It should be noted that only the samples collected in 2015 by research vessel Argos were analyzed for ^129^I. Other samples were not feasible for ^129^I analysis, since the samples have been acidified before receiving, resulting in loss of iodine due to its high volatility in acidic conditions.

## Supplementary information

Supplementary Information

## Data Availability

The data that support the findings of this study are available on request from the corresponding author upon reasonable request.

## References

[1] Steier P (2008). Natural and anthropogenic ^236^U in environmental samples. Nucl. Inst. Methods Phys. Res. B.

[2] Pollington AD, Kinman WS, Hanson SK, Steiner RE (2016). Polyatomic interferences on high precision uranium isotope ratio measurements by MC-ICP-MS: applications to environmental sampling for nuclear safeguards. J. Radioanal. Nucl. Chem..

[3] Hedberg PML, Peres P, Fernandes F, Renaud L (2015). Multiple ion counting measurement strategies by SIMS—a case study from nuclear safeguards and forensics. J. Anal. Spectrom..

[4] Ranebo Y, Hedberg PML, Whitehouse MJ, Ingeneri K, Littmann S (2009). Improved isotopic SIMS measurements of uranium particles for nuclear safeguard purposes. J. Anal. Spectrom..

[5] Bu W (2017). Development and application of mass spectrometric techniques for ultra-trace determination of ^236^U in environmental samples—a review. Anal. Chim. Acta.

[6] Christl M (2012). A depth profile of uranium-236 in the Atlantic Ocean. Geochim. Cosmochim. Acta.

[7] Qiao J (2017). Anthropogenic ^236^U in Danish seawater: global fallout versus reprocessing discharge. Environ. Sci. Technol..

[8] Sakaguchi A (2009). First results on ^236^U levels in global fallout. Sci. Total Environ..

[9] Hain, K. et al. ^233^U/^236^U signature allows to distinguish environmental emissions of civil nuclear industry from weapons fallout. *Nat. Commun*. **11**10.1038/s41467-020-15008- (2020). 10.1038/s41467-020-15008-2 PMC7062840 32152279

[10] Steier P (2019). The actinide beamline at VERA. Nucl. Instrum. Methods Phys. Res. Sect. B Beam Interact. Mater. At..

[11] HELCOM MORS Discharge database. https://helcom.fi/baltic-sea-trends/data-maps/databases/.

[12] Naegeli, R. E. Calculation of the radionuclides in PWR spent fuel samples for SFR experiment planning. Sandia National Laboratories http://prod.sandia.gov/techlib/access-control.cgi/2004/042757.pdf (2004).

[13] Casacuberta N (2016). First ^236^U data from the Arctic Ocean and use of ^236^U/^238^U and ^129^I/^236^U as a new dual tracer. Earth Planet. Sci. Lett..

[14] Castrillejo M (2018). Tracing water masses with ^129^I and ^236^U in the subpolar North Atlantic along the GEOTRACES GA01 section. Biogeosciences.

[15] Casacuberta N (2018). Tracing the three Atlantic branches entering the Arctic Ocean with ^129^I and ^236^U. J. Geophys. Res. Ocean..

[16] Wefing AM, Christl M, Vockenhuber C, Rutgers van der Loeff M, Casacuberta N (2019). Tracing Atlantic waters using ^129^I and ^236^U in the Fram Strait in 2016. J. Geophys. Res. Ocean.

[17] Jakobs, G. *Spatial and Seasonal Distribution of Methane and Its Microbial Oxidation in the Water Column of the Central Baltic Sea*. PhD thesis, University of Rostock (2014).

[18] Dahlgaard, H. Baltic 137Cs outflow through the Danish Straits indicates remobilisation, HELCOM MORS-PRO 8/2003 (2003).

[19] Andersen, J. H. et al. Development of tools for assessment of eutrophication in the Baltic Sea. Baltic Sea Environment Proceedings No. 104 (2006).

[20] Christl M, Casacuberta N, Lachner J, Herrmann J, Synal HA (2017). Anthropogenic ^236^U in the North Sea—a closer look into a source region. Environ. Sci. Technol..

[21] Yi P, Aldahan A, Hansen V, Possnert G, Hou XL (2011). Iodine isotopes (^129^I and ^127^I) in the Baltic Proper, Kattegat, and Skagerrak Basins. Environ. Sci. Technol..

[22] Aldahan, A., Kekli, A. & Possnert, G. Distribution and sources of ^129^I in rivers of the Baltic region. *J. Environ. Radioact*. 10.1016/j.jenvrad.2006.01.003 (2006). 10.1016/j.jenvrad.2006.01.003 16527378

[23] Hardy EP, Krey PW, Volchok HL (1973). Global inventory and distribution of fallout plutonium. Nature.

[24] Christl M (2015). Reconstruction of the ^236^U input function for the Northeast Atlantic Ocean: Implications for ^129^I/^236^U and ^236^U/^238^U-based tracer ages. J. Geophys. Res. Ocean.

[25] Eigl R, Steier P, Sakata K, Sakaguchi A (2017). Vertical distribution of ^236^U in the North Pacific Ocean. J. Environ. Radioact..

[26] Castrillejo M (2020). Unravelling 5 decades of anthropogenic ^236^U discharge from nuclear reprocessing plants. Sci. Total Environ..

[27] Ketterer ME, Hafer KM, Mietelski JW (2004). Resolving Chernobyl vs. global fallout contributions in soils from Poland using plutonium atom ratios measured by inductively coupled plasma mass spectrometry. J. Environ. Radioact..

[28] Andersson PS, Wasserburg GJ, Chen JH, Papanastassiou DA, Ingri J (1995). ^238^U-^234^U and ^232^Th-^230^Th in the Baltic Sea and in river water. Earth Planet. Sci. Lett..

[29] Christl M (2013). First data of Uranium-236 in the North Sea. Nucl. Instr. Meth. B.

[30] Müller JW (2000). Possible advantages of a robust evaluation of comparisons. J. Res. Natl Inst. Stand. Technol..

[31] Markus Meier, H. E. & Kauker, F. Modeling decadal variability of the Baltic Sea: 2. Role of freshwater inflow and large-scale atmospheric circulation for salinity. *J. Geophys. Res. C. Ocean* **108** (2003).

[32] Gustafsson BG, Westman P (2002). On the causes for salinity variations in the Baltic Sea during the last 8500 years. Paleoceanography.

[33] Mohrholz V, Naumann M, Nausch G, Krüger S, Gräwe U (2015). Fresh oxygen for the Baltic Sea—an exceptional saline inflow after a decade of stagnation. J. Mar. Syst..

[34] Salbu, B. & Lind, O. C. Radioactive particles released into the environment from nuclear events. *Actin. Nanoparticle Res*. 335–359 10.1007/978-3-642-11432-8 (2011).

[35] Gupta, C. *Material in Nuclear Energy Applications* (Taylor & Francis Group, 1989).

[36] Reistad, O. & Olgaard, P. L. Russian nuclear power plants for marine applications. NKS-138 (2006).

[37] Yablokov AV (2001). Radioactive waste disposal in seas adjacent to the territory of the Russian Federation. Mar. Pollut. Bull..

[38] Nielsen SP (1999). The radiological exposure of man from radioactivity in the Baltic Sea. Sci. Total Environ..

[39] IAEA. Inventory of radioactive waste disposals at sea. IAEA-TECDOC-1105 vol. 4 (1999).

[40] HELCOM. Thematic assessment of long-term changes in radioactivity in the Baltic Sea, 2007–2010. Baltic Sea Environment Proceedings No.135 (2013).

[41] Zaborska, A., Winogradow, A. & Pempkowiak, J. Caesium-137 distribution, inventories and accumulation history in the Baltic Sea sediments. *J. Environ. Radioact*. 10.1016/j.jenvrad.2013.09.003 (2014). 10.1016/j.jenvrad.2013.09.003 24121306

[42] Wullf F, Stigebrandt A, Rahm L (1990). Nutrient dynamics of the Baltic Sea. Ambio.

[43] Myrberg K, Andrejev O (2006). Modelling of the circulation, water exchange and water age properties of the Gulf of Bothnia. Oceanologia.

[44] Yi P (2013). 129I in the Baltic Proper and Bothnian Sea: application for estimation of water exchange and environmental impact. J. Environ. Radioact..

[45] Shinonaga, T., Steier, P., Lagos, M. & Ohkura, T. Airborne plutonium and non-natural uranium from the Fukushima DNPP found at 120 km distance a few days after reactor hydrogen explosions. *Environ. Sci. Technol*. **48** (2014). 10.1021/es404961w 24621142

[46] Qiao J, Hou X, Roos P, Miró M (2010). Rapid and simultaneous determination of neptunium and plutonium isotopes in environmental samples by extraction chromatography using sequential injection analysis and ICP-MS. J. Anal. Spectrom..

[47] Qiao J, Hou X, Steier P, Nielsen S, Golser R (2015). Method for ^236^U determination in seawater using flow injection extraction chromatography and accelerator mass spectrometry. Anal. Chem..

[48] Qiao, J., Hain, K. & Steier, P. First dataset of ^236^U and ^233^U around Greenland coast: a 5-year snapshot (2012–2016). *Chemosphere* **257**, 127185 (2020). 10.1016/j.chemosphere.2020.127185 32497842

[49] Lachner J, Christl M, Vockenhuber C, Synal H-A (2013). Detection of UH3+ and ThH3+ molecules and 236U background studies with low-energy AMS. Nucl. Inst. Methods Phys. Res. B.

[50] Winkler SR (2015). He stripping for AMS of ^236^U and other actinides using a 3 MV tandem accelerator. Nucl. Instrum. Methods Phys. Res. Sect. B Beam Interact. Mater. At..

[51] Lachner J (2012). Existence of triply charged actinide-hydride molecules. Phys. Rev. A Mol. Opt. Phys..

