# Supplementary material for: An unknown source of reactor radionuclides in the Baltic Sea revealed by multi-isotope fingerprints
Source: Nat Commun. 2021 Feb 5;12:823. doi: 10.1038/s41467-021-21059-w (PMC7865023; doi:10.1038/s41467-021-21059-w)

# Supplementary information

## An unknown source of reactor radionuclides in the Baltic Sea revealed by multi-isotope fingerprints

*Jixin Qiao [1*], Haitao Zhang [1, 2], Peter Steier [3], Karin Hain [3], Xiaolin Hou [1], Vesa-Pekka Vartti [4], Gideon M. Henderson [5], Mats Eriksson [6, 7], Ala Aldahan [8], Göran Possnert [9], Robin Golser [3]*

[1] Department of Environmental Engineering, Technical University of Denmark, DTU Risø Campus, Roskilde, Denmark

[2] Northwest Institute of Nuclear Technology, Xi'an, China

[3] Faculty of Physics, Isotope Physics, University of Vienna, Vienna, Austria

[4] Environmental Radiation Surveillance, Radiation and Nuclear Safety Authority, Helsinki, Finland

[5] Department of Earth Sciences, University of Oxford, Oxford, United Kingdom

[6] Department of Health, Medicine and Caring Sciences, Linköping University, Linköping, Sweden

[7] Department of Radiation Protection, Swedish Radiation Safety Authority, Stockholm, Sweden

[8] Department of Geology, United Arab Emirates University, Al Ain, United Arab Emirates

[9] Tandem Laboratory, Uppsala University, Uppsala, Sweden

[*] email: jiqi@env.dtu.dk

**Supplementary Table 1.** Overall results of salinities, concentrations of $^{238}U$, $^{236}U$, $^{127}I$ and $^{129}I$, atomic ratios of $^{236}U/^{238}U$, $^{233}U/^{236}U$, $^{129}I/^{127}I$ and $^{236}U/^{129}I$ in the Baltic Sea water

| ID | Sampling date | Location | | Region | Depth | Salinity | $^{238}U$ | $^{236}U/^{238}U$ atomic ratio | $^{236}U$ | $^{233}U/^{236}U$ atomic ratio | $^{127}I$ | $^{129}I/^{127}I$ atomic ratio | $^{129}I$ | $^{236}U/^{129}I$ atomic ratio |
|---|---|---|---|---|---|---|---|---|---|---|---|---|---|---|
| | dd-mm-yyyy | °E | °N | | m | ‰ | μg/L | $\times 10^{-9}$ | $\times 10^{7}$ atom/L | $10^{-2}$ | μg/L | $\times 10^{-9}$ | $\times 10^{9}$ atoms/L | $\times 10^{-4}$ |
| 2015-0546 | 01/07/2015 | 19.88 | 58.00 | MBR2015 | 0-5 | 7.07 | 0.719 ± 0.028 | 25.4 ± 0.8 | 4.62 ± 0.23 | 0.55 ± 0.05 | 7.25 ± 0.36 | 101 ± 8 | 3.47 ± 0.12 | 133 |
| 2015-0551 | 01/07/2015 | 20.05 | 57.33 | MBR2015 | 0-5 | 7.52 | 0.709 ± 0.020 | 28.4 ± 1.0 | 5.08 ± 0.22 | 0.43 ± 0.04 | 7.29 ± 0.37 | 116 ± 9 | 4.02 ± 0.14 | 127 |
| 2015-0556 | 01/07/2015 | 19.58 | 56.60 | MBR2015 | 0-5 | 7.70 | 0.760 ± 0.025 | 25.6 ± 0.9 | 4.91 ± 0.23 | 0.52 ± 0.15 | 8.77 ± 0.44 | 104 ± 7 | 4.31 ± 0.14 | 114 |
| 2015-0561 | 01/07/2015 | 18.40 | 55.55 | SBR2015 | 0-5 | 8.02 | 0.750 ± 0.020 | 23.8 ± 0.8 | 4.52 ± 0.19 | 0.47 ± 0.07 | 9.41 ± 0.47 | 120 ± 8 | 5.36 ± 0.18 | 84 |
| 2015-0566 | 01/07/2015 | 15.98 | 55.25 | SBR2015 | 0-5 | 8.35 | 0.791 ± 0.025 | 22.7 ± 0.8 | 4.54 ± 0.22 | 0.48 ± 0.17 | 9.41 ± 0.45 | 137 ± 9 | 6.13 ± 0.20 | 74 |
| 2015-0571 | 01/07/2015 | 15.33 | 55.38 | SB2015 | 0-5 | 8.31 | 0.750 ± 0.039 | 28.5 ± 0.9 | 5.40 ± 0.33 | 0.41 ± 0.06 | 8.44 ± 0.42 | 125 ± 9 | 4.99 ± 0.17 | 108 |
| 2015-0576 | 01/07/2015 | 14.08 | 55.80 | NBR2015 | 0-5 | 8.55 | 0.811 ± 0.026 | 21.0 ± 0.7 | 4.31 ± 0.20 | 0.49 ± 0.08 | 10.3 ± 0.5 | 147 ± 14 | 7.19 ± 0.60 | 60 |
| 2015-0577 | 02/07/2015 | 13.30 | 55.00 | NBR2015 | 0-5 | 8.91 | 0.842 ± 0.036 | 38.0 ± 1.2 | 8.09 ± 0.44 | 0.50 ± 0.17 | 9.82 ± 0.49 | 165 ± 16 | 7.67 ± 0.65 | 105 |
| 2015-0582 | 02/07/2015 | 12.75 | 55.87 | DS2015 | 0-5 | 12.3 | 1.04 ± 0.04 | 32.3 ± 1.1 | 8.48 ± 0.44 | 0.49 ± 0.04 | 14.8 ± 0.4 | 295 ± 28 | 20.7 ± 1.8 | 41 |
| 2015-0587 | 01/07/2015 | 12.12 | 56.67 | KGR2015 | 0-5 | 18.4 | 1.59 ± 0.05 | 13.2 ± 0.5 | 5.32 ± 0.24 | 0.45 ± 0.02 | 22.7 ± 0.7 | 526 ± 24 | 56.7 ± 2.3 | 9 |
| 2015-0592 | 01/07/2015 | 11.67 | 57.18 | KGR2015 | 0-5 | 20.0 | 1.72 ± 0.07 | 12.1 ± 0.4 | 5.25 ± 0.27 | 0.21 ± 0.10 | 24.9 ± 0.7 | 607 ± 26 | 71.5 ± 2.7 | 7 |
| 2015-0597 | 01/07/2015 | 10.50 | 58.27 | KGR2015 | 0-5 | 34.1 | 2.95 ± 0.11 | 7.22 ± 0.2 | 5.39 ± 0.27 | 0.34 ± 0.03 | 47.6 ± 1.0 | 219 ± 9 | 49.5 ± 1.9 | 11 |
| 2015-0602 | 01/07/2015 | 10.85 | 58.28 | KGR2015 | 0-5 | 34.2 | 2.98 ± 0.11 | 10.7 ± 0.4 | 8.09 ± 0.40 | 0.18 ± 0.03 | 47.6 ± 1.0 | 681 ± 12 | 154 ± 2 | 5 |
| 2015-0607 | 01/07/2015 | 11.03 | 58.33 | KGR2015 | 0-5 | 21.8 | 1.86 ± 0.62 | 10.7 ± 0.3 | 5.03 ± 0.23 | 0.32 ± 0.04 | 27.0 ± 0.8 | 454 ± 10 | 58.2 ± 0.8 | 9 |
| 2015-0612 | 01/07/2015 | 11.43 | 58.25 | KGR2015 | 0-5 | 23.7 | 1.99 ± 0.10 | 10.7 ± 0.4 | 5.37 ± 0.32 | 0.35 ± 0.05 | 29.6 ± 0.9 | 502 ± 11 | 70.5 ± 0.9 | 8 |
| 2015-0617 | 01/07/2015 | 11.30 | 57.87 | KGR2015 | 0-5 | 31.7 | 2.83 ± 0.07 | 14.6 ± 0.5 | 10.5 ± 0.43 | 0.22 ± 0.02 | 38.1 ± 1.1 | 1290 ± 28 | 232 ± 3.9 | 5 |
| 2015-0622 | 01/07/2015 | 12.20 | 56.93 | KGR2015 | 0-5 | 19.3 | 1.68 ± 0.04 | 11.6 ± 0.4 | 4.96 ± 0.20 | 0.44 ± 0.04 | 22.7 ± 0.7 | 541 ± 15 | 58.3 ± 1.0 | 9 |
| 2015-0627 | 02/07/2015 | 14.87 | 55.62 | SBR2015 | 0-5 | 8.36 | 0.750 ± 0.031 | 32.3 ± 1.0 | 6.13 ± 0.30 | 0.35 ± 0.03 | 8.39 ± 0.42 | 157 ± 10 | 6.23 ± 0.12 | 98 |
| 2015-0632 | 01/07/2015 | 16.20 | 56.37 | MBR2015 | 0-5 | 7.86 | 0.688 ± 0.030 | 24.3 ± 0.8 | 4.23 ± 0.23 | 0.82 ± 0.09 | 8.49 ± 0.38 | 148 ± 9 | 5.96 0.14 | 71 |
| 2015-0636 | 01/07/2015 | 17.07 | 57.12 | MBR2015 | 0-5 | 7.42 | 0.688 ± 0.021 | 27.4 ± 0.9 | 4.78 ± 0.22 | 0.44 ± 0.06 | 7.92 ± 0.39 | 124 ± 8 | 4.65 0.11 | 103 |
| 2015-0641 | 01/07/2015 | 17.98 | 58.02 | MBR2015 | 0-5 | 6.77 | 0.657 ± 0.024 | 26.3 ± 0.9 | 4.37 ± 0.22 | 0.50 ± 0.03 | 6.72 ± 0.32 | 109 ± 9 | 3.47 0.09 | 126 |
| 2015-0463 | 15/7/2015 | 15.15 | 55.08 | SBR2015 | 0-5 | 7.85 | 1.02 ± 0.04 | 16.4 ± 0.6 | 4.21 ± 0.23 | 0.67 ± 0.17 | | | | |
| 2014-0413 | 01/07/2014 | 12.37 | 56.20 | DS2014 | 0-5 | 12.8 | 1.36 ± 0.04 | 13.5 ± 2.3 | 4.64 ± 0.80 | | | | | |
| 2014-0415 | 01/07/2014 | 11.78 | 56.17 | DS2014 | 0-5 | 15.9 | 1.95 ± 0.06 | 15.9 ± 1.9 | 7.83 ± 0.96 | | | | | |
| 2014-0417 | 01/07/2014 | 11.17 | 56.12 | DS2014 | 0-5 | 15.8 | 1.68 ± 0.06 | 9.7 ± 1.3 | 4.11 ± 0.58 | | | | | |
| 2014-0419 | 01/07/2014 | 10.77 | 55.65 | DS2014 | 0-5 | 14.6 | 1.57 ± 0.05 | 19.7 ± 1.8 | 7.84 ± 0.75 | | | | | |
| 2014-0421 | 01/07/2014 | 11.05 | 55.38 | DS2014 | 0-5 | 13.1 | 1.47 ± 0.05 | 15.3 ± 1.6 | 5.67 ± 0.60 | | | | | |
| 2014-0423 | 01/07/2014 | 10.83 | 54.87 | DS2014 | 0-5 | 13.6 | 1.52 ± 0.05 | 11.9 ± 1.1 | 4.57 ± 0.47 | | | | | |
| 2014-0425 | 01/07/2014 | 11.05 | 54.65 | DS2014 | 0-5 | 12.7 | 1.56 ± 0.05 | 19.0 ± 1.7 | 7.48 ± 0.71 | | | | | |
| 2014-0427 | 01/07/2014 | 11.98 | 54.47 | DS2014 | 0-5 | 9.03 | 1.05 ± 0.03 | 31.6 ± 2.9 | 8.38 ± 0.80 | | | | | |
| 2014-0429 | 01/07/2014 | 12.68 | 54.95 | SBR2014 | 0-5 | 8.61 | 0.969 ± 0.029 | 24.0 ± 2.5 | 5.88 ± 0.63 | | | | | |
| 2014-0431 | 01/07/2014 | 12.60 | 55.42 | DS2014 | 0-5 | 8.31 | 0.943 ± 0.029 | 23.3 ± 3.0 | 5.56 ± 0.74 | | | | | |
| 2014-0433 | 01/07/2014 | 12.73 | 55.80 | DS2014 | 0-5 | 9.69 | 1.05 ± 0.03 | 13.8 ± 1.4 | 3.65 ± 0.38 | | | | | |
| 2014-0435 | 01/07/2014 | 12.00 | 56.66 | KGR2014 | 0-5 | 16.6 | 1.89 ± 0.06 | 9.34 ± 1.50 | 4.46 ± 0.73 | | | | | |
| 2014-0600 | 01/07/2014 | 11.99 | 55.96 | DS2014 | 0-5 | 19.7 | 2.09 ± 0.06 | 11.8 ± 1.1 | 6.25 ± 0.61 | | | | | |
| 2014-0601 | 01/07/2014 | 11.93 | 55.95 | DS2014 | 0-5 | 22.0 | 2.05 ± 0.06 | 14.8 ± 1.6 | 7.71 ± 0.86 | | | | | |
| 2014-0602 | 01/07/2014 | 12.02 | 55.96 | DS2014 | 0-5 | - | 2.14 ± 0.07 | 12.6 ± 1.2 | 6.81 ± 0.67 | | | | | |
| 2014-0603 | 01/07/2014 | 12.02 | 55.70 | DS2014 | 0-5 | 15.7 | 1.90 ± 0.06 | 10.2 ± 1.3 | 4.89 ± 0.62 | | | | | |
| 2014-0604 | 01/07/2014 | 11.85 | 55.94 | DS2014 | 0-5 | - | 2.27 ± 0.07 | 13.4 ± 1.2 | 7.68 ± 0.74 | | | | | |
| 2014-0605 | 01/07/2014 | 12.09 | 55.70 | DS2014 | 0-5 | - | 1.90 ± 0.06 | 13.3 ± 1.3 | 6.39 ± 0.63 | | | | | |
| 2014-0606 | 01/07/2014 | 12.02 | 55.72 | DS2014 | 0-5 | - | 1.90 ± 0.06 | 9.45 ± 0.99 | 4.55 ± 0.50 | | | | | |
| 2014-0607 | 01/07/2014 | 12.07 | 55.74 | DS2014 | 0-5 | - | 1.97 ± 0.06 | 11.3 ± 1.5 | 5.64 ± 0.75 | | | | | |
| 2014-0608 | 01/07/2014 | 11.85 | 55.94 | DS2014 | 0-5 | - | 1.97 ± 0.06 | 11.3 ± 1.5 | 5.64 ± 0.75 | | | | | |
| 2013-0508 | 21/06/2013 | 10.28 | 56.81 | KGR2013 | 0-5 | 26.0 | 2.39 ± 0.07 | 5.47 ± 0.50 | 3.31 ± 0.32 | 0.14 ± 0.05 | | | | |
| 2013-0509 | 21/06/2013 | 10.52 | 57.35 | KGR2013 | 0-5 | 28.4 | 2.72 ± 0.08 | 9.10 ± 0.83 | 6.27 ± 0.60 | 0.22 ± 0.05 | | | | |
| 2013-0510 | 22/06/2013 | 10.52 | 57.73 | KGR2013 | 0-5 | 29.7 | 2.88 ± 0.09 | 6.92 ± 0.66 | 5.04 ± 0.50 | 0.21 ± 0.04 | | | | |
| 2013-0511 | 22/06/2013 | 9.99 | 57.59 | KGR2013 | 0-5 | 30.5 | 2.93 ± 0.09 | 5.83 ± 0.53 | 4.32 ± 0.41 | 0.20 ± 0.06 | | | | |
| 2013-0512 | 22/06/2013 | 8.63 | 57.12 | KGR2013 | 0-5 | 32.1 | 3.00 ± 0.09 | 9.34 ± 0.86 | 7.09 ± 0.69 | 0.22 ± 0.06 | | | | |
| 2013-0513 | 23/06/2013 | 8.22 | 56.71 | KGR2013 | 0-5 | 32.5 | 3.04 ± 0.09 | 16.2 ± 1.6 | 12.5 ± 1.25 | 0.20 ± 0.03 | | | | |
| 2013-0514 | 23/06/2013 | 8.12 | 56.00 | KGR2013 | 0-5 | 32.0 | 2.31 ± 0.07 | 15.3 ± 1.5 | 8.93 ± 0.90 | 0.20 ± 0.04 | | | | |
| 2016-0463 | 23/06/2016 | 15.15 | 55.08 | SBR2016 | 0-5 | 7.66 | 1.19 ± 0.05 | 14.5 ± 1.5 | 4.38 ± 0.24 | 0.46 ± 0.09 | | | | |
| IT039-16 | 5/29/2016 | 19.73 | 61.07 | NBR2016 | 0-5 | 5.39 | 1.07 ± 0.03 | | | | | | | |
| IT041-16 | 6/8/2016 | 20.05 | 57.32 | MBR2016 | 0-5 | 6.92 | 1.22 ± 0.03 | | | | | | | |
| IT043-16 | 6/9/2016 | 21.08 | 59.03 | MBR2016 | 0-5 | 6.37 | 1.16 ± 0.03 | | | | | | | |
| IT045-16 | 6/1/2016 | 19.97 | 62.59 | NBR2016 | 0-5 | 5.19 | 1.06 ± 0.03 | 34.4 ± 1.6 | 9.28 ± 0.50 | 0.61 ± 0.16 | | | | |
| IT047-16 | 5/31/2016 | 23.56 | 65.23 | NBR2016 | 0-5 | 2.34 | 0.824 ± 0.020 | 30.9 ± 1.9 | 6.44 ± 0.42 | 0.53 ± 0.20 | | | | |
| US-09-0920 | 11/9/2011 | 21.59 | 59.28 | MBR2011 | 0-5 | 7.32 | 0.719 ± 0.031 | 25.8 ± 3.7 | 4.69 ± 0.70 | 0.37 ± 0.08 | | | | |
| US-09-1859 | 11/9/2011 | 19.80 | 59.78 | MBR2011 | 0-5 | 5.20 | 0.544 ± 0.026 | 52.4 ± 1.0 | 7.22 ± 0.37 | 0.46 ± 0.06 | | | | |
| US-10-1347 | 11/10/2011 | 19.35 | 58.91 | MBR2011 | 0-5 | 6.56 | 0.688 ± 0.038 | 32.1 ± 1.5 | 5.58 ± 0.40 | 0.58 ± 0.10 | | | | |
| US-04-2210 | 11/4/2011 | 18.76 | 56.8 | MBR2011 | 0-5 | 7.45 | 0.750 ± 0.027 | 32.9 ± 2.1 | 6.24 ± 0.46 | 0.87 ± 0.16 | | | | |
| US-09-1519 | 11/9/2011 | 21.04 | 59.51 | MBR2011 | 0-5 | 6.29 | 0.729 ± 0.021 | 29.6 ± 3.6 | 5.46 ± 0.68 | 0.69 ± 0.18 | | | | |
| US-09-1727 | 11/9/2011 | 20.28 | 59.61 | MBR2011 | 0-5 | 5.58 | 0.637 ± 0.027 | 42.2 ± 6.9 | 6.80 ± 1.15 | 0.59 ± 0.23 | | | | |
| US-09-2059 | 11/9/2011 | 19.35 | 60.08 | NBR2011 | 0-5 | 5.96 | 0.616 ± 0.020 | 32.4 ± 4.4 | 5.05 ± 0.71 | 0.80 ± 0.16 | | | | |
| US-10-0708 | 11/10/2011 | 19.67 | 59.78 | MBR2011 | 0-5 | 5.09 | 0.575 ± 0.024 | 35.7 ± 1.7 | 5.19 ± 0.32 | 0.54 ± 0.09 | | | | |
| US-10-0930 | 11/10/2011 | 19.87 | 59.48 | MBR2011 | 0-5 | 5.81 | 0.606 ± 0.021 | 42.3 ± 1.7 | 6.49 ± 0.35 | 0.46 ± 0.11 | | | | |
| US-10-1141 | 11/10/2011 | 19.35 | 59.19 | MBR2011 | 0-5 | 6.70 | 0.760 ± 0.039 | 28.8 ± 1.6 | 5.54 ± 0.42 | 0.80 ± 0.20 | | | | |
| US-10-2335 | 11/10/2011 | 17.73 | 57.48 | MBR2011 | 0-5 | 6.48 | 0.698 ± 0.035 | 34.5 ± 2.5 | 6.10 ± 0.54 | 0.64 ± 0.18 | | | | |
| US-12-1110 | 11/12/2011 | 19.03 | 54.65 | SBR2011 | 0-5 | 6.69 | 0.739 ± 0.034 | 29.6 ± 2.0 | 5.54 ± 0.45 | 0.76 ± 0.10 | | | | |
| IT040-16 | 5/29/2016 | 19.73 | 61.07 | NBR2016 | 130 | 6.35 | 1.20 ± 0.03 | | | | | | | |
| IT042-16 | 6/8/2016 | 20.05 | 57.32 | MBR2016 | 237 | 13.8 | 1.94 ± 0.06 | | | | | | | |
| IT044-16 | 6/9/2016 | 21.08 | 59.03 | MBR2016 | 170 | 11.4 | 1.45 ± 0.04 | | | | | | | |
| IT046-16 | 6/1/2016 | 19.97 | 62.59 | NBR2016 | 220 | 6.18 | 1.28 ± 0.04 | 23.3 ± 1.0 | 7.53 ± 0.41 | 0.73 ± 0.16 | | | | |
| IT048-16 | 5/31/2016 | 23.56 | 65.23 | NBR2016 | 67 | 3.03 | 0.871 ± 0.020 | 33.4 ± 3.4 | 7.37 ± 0.77 | | | | | |

Uncertainties are expanded uncertainties using a coverage factor of k=1.

**Supplementary Table 2.** Concentration of $^{236}U$ and atomic ratios of $^{236}U/^{238}U$ and $^{233}U/^{236}U$ in Lake Mälaren and the Baltic Sea sediments

| Sample ID | Sampling date | Location | | Region | Depth | $^{236}U/^{238}U$ atomic ratio | $^{236}U$ concentration | | $^{233}U/^{236}U$ atomic ratio |
|---|---|---|---|---|---|---|---|---|---|
| | dd-mm-yyyy | °N | °E | | | | atom/L | atom/kg | |
| Lake Mälaren (n=4) | 30/04/2017 | 59.33 | 18.04 | MBR | 0-1 m | $(1.71 \pm 0.51) \times 10^{-8}$ | $(6.48 \pm 2.37) \times 10^{7}$ | | $(0.18 \pm 0.05) \times 10^{-2}$ |
| Studsvik sediment (n=3) | 25/03/2014 | 58.75 | 17.40 | MBR | 0-10 cm | $(1.35 \pm 0.17) \times 10^{-6}$ | | $(2.06 \pm 0.06) \times 10^{13}$ | $(0.36 \pm 0.05) \times 10^{-2}$ |
| Baltic sediment BY15 | 05/06/2015 | 57.32 | 20.05 | MBR | 0-2 cm | $(2.27 \pm 0.01) \times 10^{-8}$ | | $(7.99 \pm 0.80) \times 10^{11}$ | $(0.59 \pm 0.01) \times 10^{-2}$ |
| Baltic sediment LL17 | 08/06/2015 | 59.03 | 21.08 | MBR | 0-2 cm | $(2.31 \pm 0.09) \times 10^{-8}$ | | $(1.32 \pm 0.13) \times 10^{12}$ | $(0.61 \pm 0.01) \times 10^{-2}$ |
| Baltic sediment LL3a | 20/05/2015 | 60.07 | 26.33 | NBR | 0-2 cm | $(1.70 \pm 0.03) \times 10^{-8}$ | | $(1.68 \pm 0.17) \times 10^{11}$ | $(0.71 \pm 0.07) \times 10^{-2}$ |
| Baltic sediment EB1 | 26/05/2015 | 61.07 | 19.73 | NBR | 0-2 cm | $(1.36 \pm 0.09) \times 10^{-8}$ | | $(1.36 \pm 0.16) \times 10^{11}$ | - |
| Baltic sediment CVI | 28/05/2015 | 65.23 | 23.57 | NBR | 0-2 cm | $(2.47 \pm 0.05) \times 10^{-8}$ | | $(3.47 \pm 0.35) \times 10^{11}$ | $(0.83 \pm 0.03) \times 10^{-2}$ |

MBR: Middle Baltic Sea region including Northern Baltic Proper, Western Gotland Basin, Eastern Gotland Basin and Gulf of Riga; and NBR: North Baltic Sea region including Archipelago and Åland Sea, Bothnian Sea and Bothnian Bay

“n” refers to the number of replicate for the sample.

Uncertainties are expanded uncertainties using a coverage factor of k=1.

**Supplementary Table 3.** Parameters of the endmembers used in the binary mixing models in Figure 4

| Endmember | | Salinity | $^{238}U$ concentration | $^{236}U$ concentration | $^{236}U/^{238}U$ atomic ratio | $^{233}U/^{236}U$ atomic ratio | $^{233}U$ concentration | $^{233}U/^{238}U$ atomic ratio |
|---|---|---|---|---|---|---|---|---|
| | | ‰ | µg/L | $\times 10^{7}$, atom/L | $\times 10^{-8}$ | $\times 10^{-2}$ | $\times 10^{-5}$, atom/L | $\times 10^{-10}$ |
| **1** | **North Sea** | 35 | 3.3 | 8.00 | 0.958 | 0.12 | 0.960 | 0.115 |
| **2** | **Fresh water, no $^{236}U$ (L1)** | 0 | 0.4 | 0 | 0 | 1.4 | 0 | 0 |
| **2** | **Freshwater with only global fallout $^{236}U$ (L2)** | 0 | 0.4 | 3.56 ± 0.39 | 3.52 ± 0.39 | 1.4 | 4.98 ± 0.55 | 4.92 ± 0.54 |
| **2** | **Fresh water with only global fallout $^{236}U$ and best-fit $^{236}U/^{238}U$ (L)** | 0 | 0.4 | 6.87 ± 0.76 | 6.79 ± 0.75 | 1.4 | 9.62 ± 1.06 | 9.50 ± 1.05 |

**Supplementary Table 4.** Discharges of uranium isotopes from different nuclear facilities*

| Radionuclides | ABB Atom** (2002-2017), Bq | Studsvik (2011-2017), Bq | Westinghouse (1998-2017), Bq | La Hague reprocessing plant (1966-1996), Bq | Forsmark NPP (2011-2017), Bq | Oskarshamn NPP (1991), Bq | Sellafield reprocessing plant, Bq |
|---|---|---|---|---|---|---|---|
| $^{232}U$ | | | | $2.48 \times 10^{9}$ | | | |
| $^{233}U$ | $1.21 \times 10^{7}$ | | $1.21 \times 10^{7}$ | $2.69 \times 10^{6}$ | | | |
| $^{234}U$ | $2.19 \times 10^{9}$ | $1.71 \times 10^{6}$ | $1.11 \times 10^{9}$ | $3.86 \times 10^{11}$ | | | |
| $^{235}U$ | $9.84 \times 10^{7}$ | | $4.93 \times 10^{7}$ | $8.70 \times 10^{9}$ | $7.92 \times 10^{5}$ | | |
| $^{236}U$ | $1.06 \times 10^{6}$ | | $1.06 \times 10^{6}$ | $5.00 \times 10^{10}$ | | | $(1.48 \pm 0.72) \times 10^{11}$ |
| $^{238}U$ | $4.50 \times 10^{8}$ | $3.67 \times 10^{5}$ | $2.28 \times 10^{8}$ | $2.20 \times 10^{11}$ | $2.41 \times 10^{5}$ | $4.40 \times 10^{4}$ | |

*Data obtained from HELCOM MORS Discharge database. https://helcom.fi/baltic-sea-trends/data-maps/databases/

**ABB Atom was merged into Westinghouse in 2006.

**Supplementary Fig. 1 Deviation of $^{236}U/^{238}U$ and $^{236}U$ concentration from L1.** Deviations of $^{236}U/^{238}U$ atomic ratio and $^{236}U$ concertation from binary mixing line L1 (A1 and A2) and their respective geographical distributions on the map (B1 and B2). KGR: Kattegat-Skagerrak region including the Kattegat, Skagerrak and Danish west coast nearby the North Sea. DS: Danish Straits including the Belt Seas and the Sound. SBR: South Baltic Sea region including Arkona Basin, Borholm Basin and South Baltic Proper. MBR: Middle Baltic Sea region including Northern Baltic Proper, Western Gotland Basin, Eastern Gotland Basin and Gulf of Riga. NBR: North Baltic Sea region including Archipelago and Åland Sea, Bothnian Sea and Bothnian Bay.

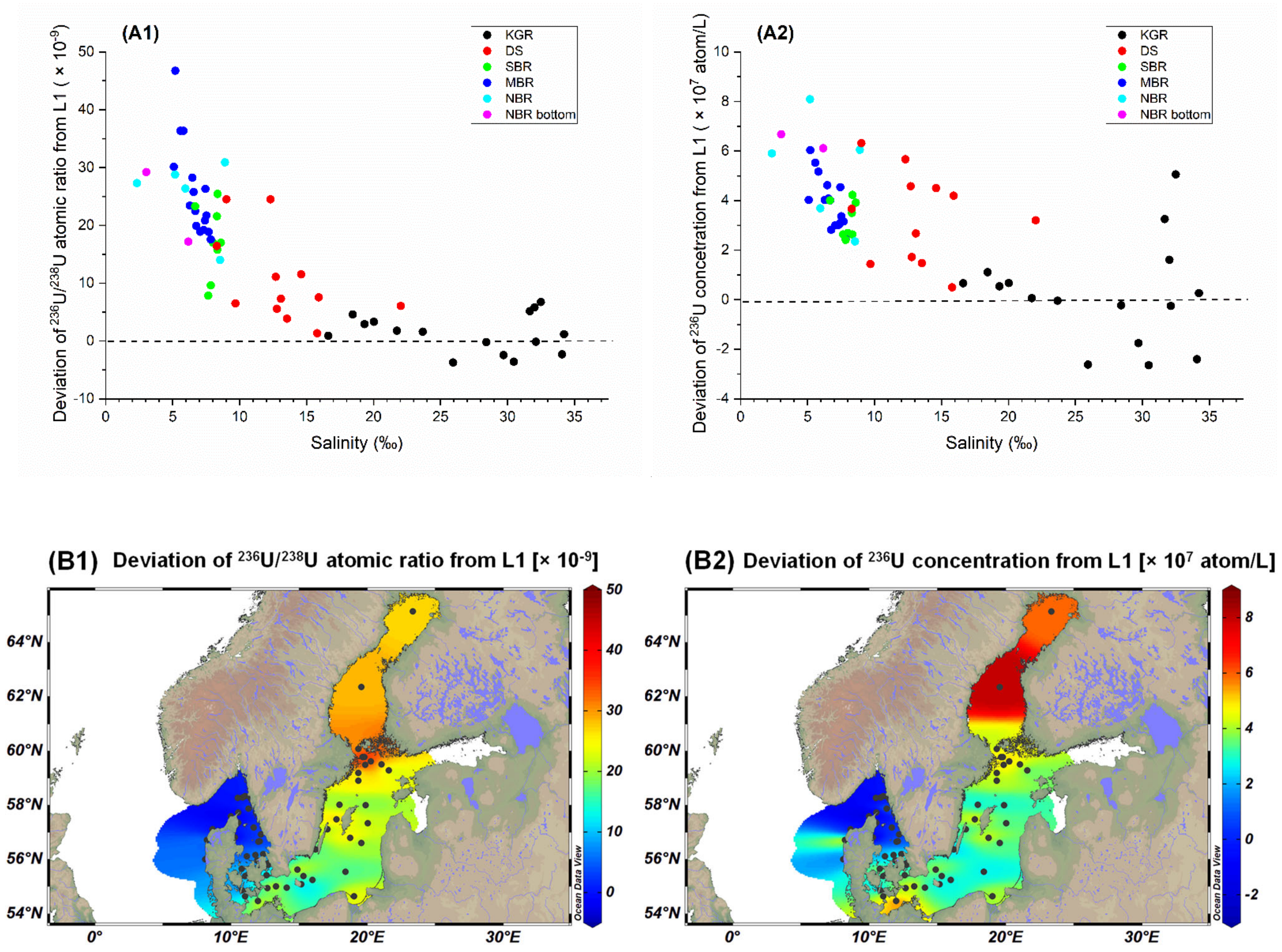

**Supplementary Fig. 2 Variation of $^{233}U/^{238}U$ with salinity.** Simulation of the freshwater endmember in the binary mixing line L2 (red dashed line). The simulation was based on $^{233}U/^{238}U$ atomic ratio vs. salinity to match the median $^{233}U/^{238}U$ atomic ratio ($(1.70 \pm 0.18) \times 10^{-10}$) for the cluster of samples from SBR, MBR and NBR at their median salinity ((6.92 ±0.29) ‰). KGR: Kattegat-Skagerrak region including the Kattegat, Skagerrak and Danish west coast nearby the North Sea. DS: Danish Straits including the Belt Seas and the Sound. SBR: South Baltic Sea region including Arkona Basin, Borholm Basin and South Baltic Proper. MBR: Middle Baltic Sea region including Northern Baltic Proper, Western Gotland Basin, Eastern Gotland Basin and Gulf of Riga. NBR: North Baltic Sea region including Archipelago and Åland Sea, Bothnian Sea and Bothnian Bay. The obtained freshwater endmember in L2: salinity =0, $^{238}U$ = 0.4 μg /L, $^{233}U/^{236}U$=1.4, $^{233}U/^{238}U=(4.92 \pm 0.54) \times 10^{-10}$, $^{236}U = (3.56 \pm 0.39) \times 10^{7}$ atom/L, and $^{236}U/^{238}U$ atomic ratio = $(3.52 \pm 0.39) \times 10^{-8}$. Uncertainties are expanded uncertainties using a coverage factor of k=1.

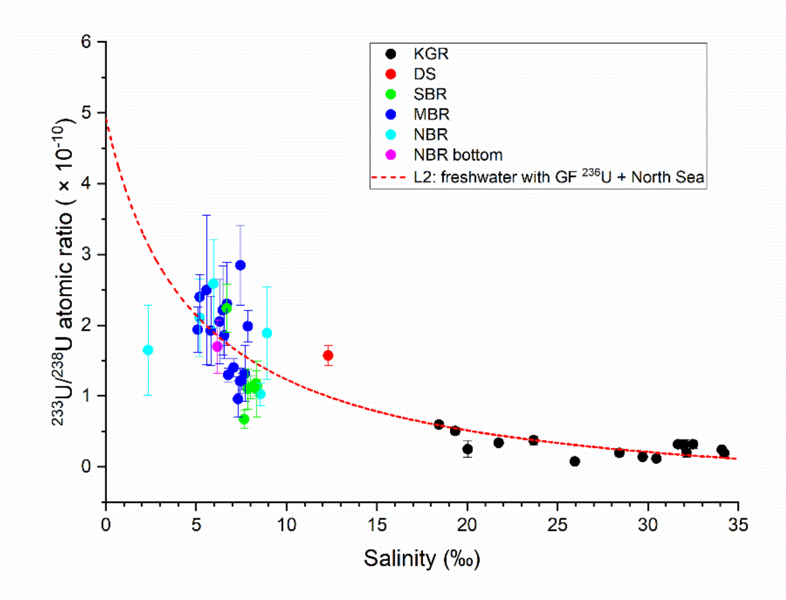

Supplement: Supplementary file 1 — Supplementary Information [file 41467_2021_21059_MOESM1_ESM.pdf]
